# Clearance of peripheral nerve misfolded mutant protein by infiltrated macrophages correlates with motor neuron disease progression

**DOI:** 10.1038/s41598-021-96064-6

**Published:** 2021-08-12

**Authors:** Wataru Shiraishi, Ryo Yamasaki, Yu Hashimoto, Senri Ko, Yuko Kobayakawa, Noriko Isobe, Takuya Matsushita, Jun-ichi Kira

**Affiliations:** 1 Department of Neurology, Neurological Institute, Graduate School of Medical Sciences, Kyushu University, 3-1-1 Maidashi, Higashi-ku, Fukuoka, 812-8582 Japan; 2 Department of Neurology, Kokura Memorial Hospital, Fukuoka, 802-8555 Japan; 3 Translational Neuroscience Center, Graduate School of Medicine, and School of Pharmacy At Fukuoka, International University of Health and Welfare, 137-1 Enokizu, Ookawa, Fukuoka 831-8501 Japan; 4 Department of Neurology, Brain and Nerve Center, Fukuoka Central Hospital, International University of Health and Welfare, 2-6-11 Yakuin, Chuo-ku, Fukuoka, 810-0022 Japan

**Keywords:** Amyotrophic lateral sclerosis, Animal disease models, Diseases of the nervous system, Glial biology, Neuroimmunology, Peripheral nervous system

## Abstract

Macrophages expressing C–C chemokine receptor type 2 (CCR2) infiltrate the central and peripheral neural tissues of amyotrophic lateral sclerosis (ALS) patients. To identify the functional role of CCR2^+^ macrophages in the pathomechanisms of ALS, we used an ALS animal model, *mutant Cu/Zn superoxide dismutase 1*^*G93A*^ (*mSOD1*)-transgenic (Tg) mice. To clarify the CCR2 function in the model, we generated *SOD1*^G93A^/*CCR2*^*Red fluorescence protein (*RFP)/Wild type *(WT)*^/*CX3CR1*^*Green fluorescence protein (GFP)/WT*^-Tg mice, which heterozygously express *CCR2-RFP* and *CX3CR1-GFP*, and *SOD1*^G93A^/*CCR2*^RFP/RFP^-Tg mice, which lack CCR2 protein expression and present with a CCR2-deficient phenotype. In *mSOD1*-Tg mice, mSOD1 accumulated in the sciatic nerve earlier than in the spinal cord. Furthermore, spinal cords of *SOD1*^G93A^/*CCR2*^RFP/WT^/*CX3CR1*^GFP/WT^ mice showed peripheral macrophage infiltration that emerged at the end-stage, whereas in peripheral nerves, macrophage infiltration started from the pre-symptomatic stage. Before disease onset, CCR2^+^ macrophages harboring mSOD1 infiltrated sciatic nerves earlier than the lumbar cord. CCR2-deficient *mSOD1*-Tg mice showed an earlier onset and axonal derangement in the sciatic nerve than CCR2-positive *mSOD1*-Tg mice. CCR2-deficient *mSOD1*-Tg mice showed an increase in deposited mSOD1 in the sciatic nerve compared with CCR2-positive mice. These findings suggest that CCR2^+^ and CX3CR1^+^ macrophages exert neuroprotective functions in mSOD1 ALS via mSOD1 clearance from the peripheral nerves.

## Introduction

Amyotrophic lateral sclerosis (ALS) is a fatal neurodegenerative disease characterized by the loss of upper and lower motor neurons. Although the majority of ALS cases are sporadic, approximately 10% are inherited^1^. Sporadic and familial ALS are clinically and pathologically similar. Approximately 20% of familial cases are linked to autosomal dominant mutations in the Cu/Zn superoxide dismutase 1 (*SOD1*) gene^2^. Hallmark pathological features in sporadic and familial ALS include the presence of axonal spheroids and perikaryal accumulation of inclusion bodies comprising neuronal intermediate filament proteins, such as neurofilaments and peripherin^3,4^.

Although the exact mechanism of ALS remains elusive, protein misfolding and aggregation have been implicated as contributing factors to motor neuron death^5^. This abnormal protein aggregation is thought to trigger non-cell autonomous neuronal cell death via glia-mediated mechanisms^6^. In ALS, activated microglia, macrophages, and astrocytes may be neuroprotective in the early stage but become pro-inflammatory and neurotoxic in the later stage when damage-associated molecular patterns are released from injured motoneurons with accumulated misfolded proteins, and they promote the pro-inflammatory activation of glial cells^7,8^. We and others have reported a variety of immune abnormalities in ALS, including increased cerebrospinal fluid (CSF) pro-inflammatory cytokines/chemokines, such as interleukin (IL)-1β, IL-12, IL-17, tumor necrosis factor-α, interferon-γ, C–C motif chemokine ligand (CCL) 2, CCL4, CCL11, C-X-C motif chemokine ligand (CXCL) 8, and CXCL10^9^, increased serum IL-6, IL-17, and CCL2^10–12^, and increased circulating IL-13-producing T cells^13^. However, it remains unclear whether these immune abnormalities cause disease or are a consequence of disease and whether they are neurotoxic or neuroprotective according to the disease stage.

Monocyte-macrophage lineage cells are heterogenous and express distinct chemokine receptors^14^. C–C chemokine receptor 2 (CCR2) is expressed by peripheral monocytes/macrophages, in addition to T cells, basophils, and immature dendritic cells^15^, whereas resident macrophages, such as microglia in the central nervous system (CNS), Kupffer cells in the liver, and Langerhans cells in the skin, express high levels of C-X3-C chemokine receptor 1 (CX3CR1)^16^. Notably, a CCL2/CCR2-dependent immunological pathway has been implicated in ALS. Patients had an increased level of CCL2 in the CSF and serum about 1 year before the onset of ALS^9,17^. Furthermore, spinal cord tissues from mutant SOD1 (mSOD1) transgenic ALS model mice also showed an increased level of CCL2 mRNA^18,19^. Conversely, CCR2 expression in the peripheral blood monocytes of ALS patients was significantly decreased compared with healthy controls^20,21^. These results collectively suggest the initial recruitment of peripheral monocytes/macrophages expressing CCR2 to neural tissues. However, it remains to be established whether these monocytes/macrophages exert protective or deleterious effects in ALS pathogenesis.

To address this issue in the present study, we aimed to clarify the functions of CCR2-bearing monocytes/macrophages recruited from the peripheral blood to the neural tissues in ALS. For this purpose, genetically labeled transgenic “Red-Green” *SOD1*^G93A^ mice, which express *CCR2-red fluorescence protein (RFP)* and *CX3CR1-green fluorescence protein (GFP)* heterozygously, were used to characterize monocyte lineage cell dynamics. Furthermore, the role of CCR2 in ALS was explored using *SOD1*^G93A^/*CCR2*^RFP/RFP^ mice, which possess a CCR2-deficient phenotype.

## Results

### mSOD1 accumulates in peripheral nerves earlier than in the spinal cord

**Figure 1:**
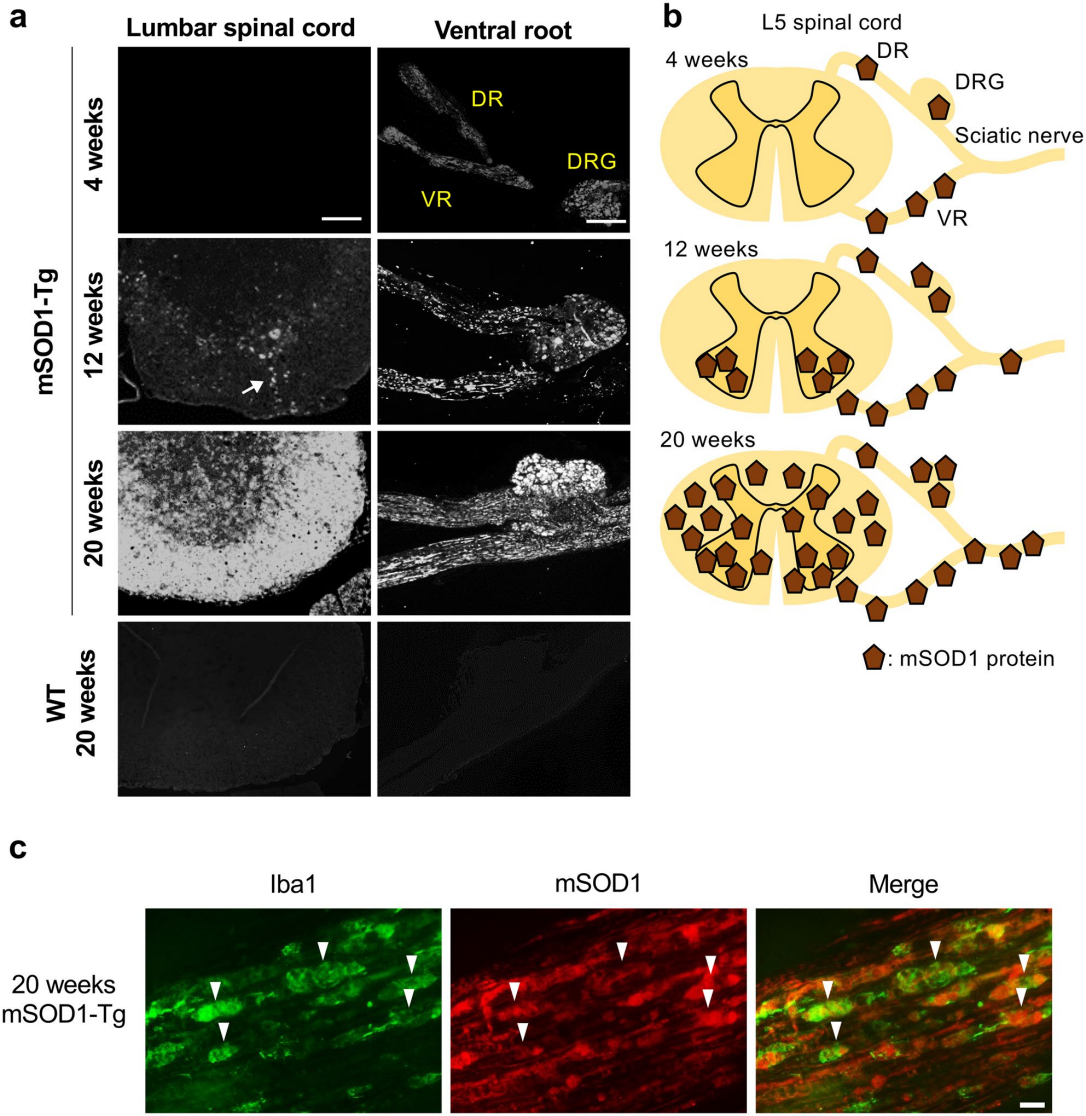
Immunostaining for mSOD1 and Iba1 in neural tissues. (**a**) Immunostaining for human mSOD1 in the lumbar spinal cord, dorsal root ganglia (DRG), ventral root (VR), and dorsal root (DR) from *CCR2*^RFP/WT^/*CX3CR1*^GFP/WT^ (non-mSOD1 type) and *SOD1*^G93A^/*CCR2*^RFP/WT^/*CX3CR1*^GFP/WT^ (mSOD1-Tg) mice. Accumulation of mSOD1 protein in the DRG, VR, and DR prior to accumulation in the lumbar cord was apparent at 4 weeks of age. In the spinal cord, mSOD1 protein started to accumulate along the intramedullary lower motoneuron axonal tracts (arrow) at 12 weeks of age. (**b**) Schemas depict mSOD1 protein accumulation over the disease course in the mSOD1-Tg ALS mouse model. (**c**) Double immunostaining for Iba1 (green) and mSOD1 (red) in the sciatic nerves of a 20-week-old mSOD1-Tg mouse. All Iba1^+^ macrophages (arrowhead) harbored mSOD1 protein. Scale bars: (**a**) 200 μm (lumbar spinal cord), and 100 μm (DRG, VR, and DR): (**c**) 10 μm.

mSOD1 immunostaining of *SOD1*^G93A^ mice indicated the deposition of mSOD1 was absent in the spinal cord at 4 weeks of age and mainly present in the anterior horns at 12 weeks of age (onset stage) (Fig. 1a, b). mSOD1 protein was accumulated and spread along the pyramidal tracts and eventually extended into whole spinal cord cross-sectional areas at 20 weeks of age (moribund period). Conversely, the accumulation of mSOD1 in peripheral nervous system (PNS) tissues was detected in the spinal roots and dorsal root ganglia (DRG) as early as 4 weeks of age and was increased abundantly in the ventral roots compared with the dorsal roots. Double immunostaining for Iba1 and mSOD1 demonstrated Iba1^+^ foamy macrophages in the sciatic nerve were filled with mSOD1 protein at 20 weeks of age (Fig. 1c).

### Microglial responses commence in the spinal anterior horns at clinical disease onset

**Figure 2:**
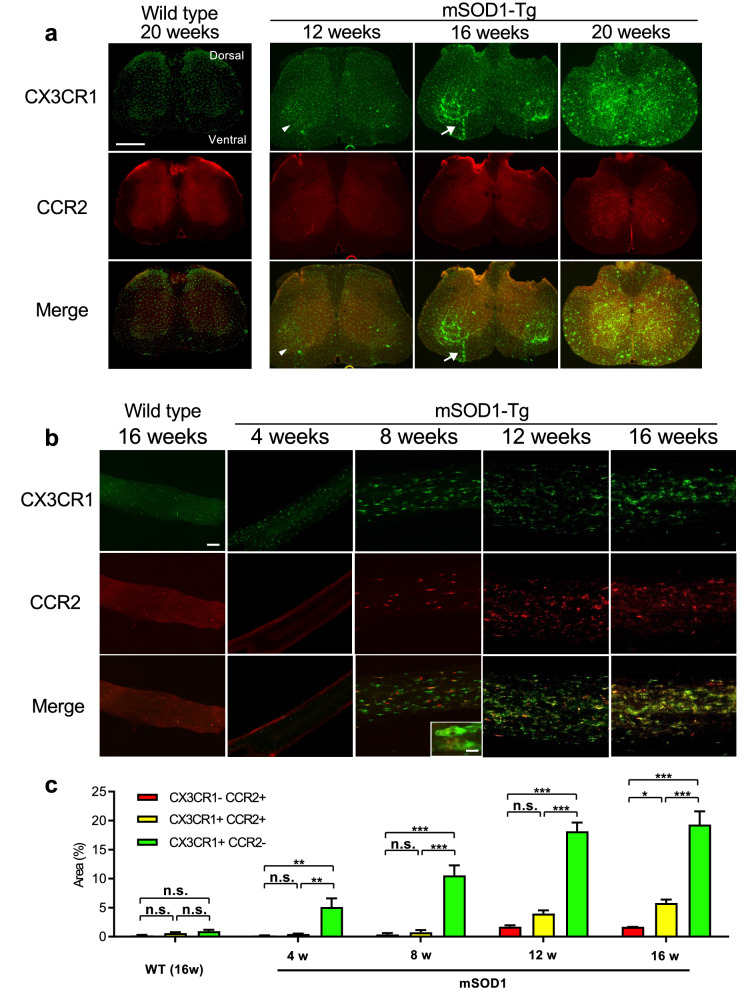
CX3CR1 and CCR2 expression in the lumbar spinal cord and sciatic nerve. (**a**) Low magnification fluorescence images of *CCR2*^RFP/WT^/*CX3CR1*^GFP/WT^ (non-mSOD1 type) and *SOD1*^G93A^/*CCR2*^RFP/WT^/*CX3CR1*^GFP/WT^ (mSOD1-Tg) mouse spinal cord. At 12 weeks of age, CX3CR1^+^ (green) but not CCR2^+^ (red) cells were sparsely visible only in the spinal anterior horns (arrowhead). CX3CR1^+^ cells markedly increased as the disease progressed from 16 to 20 weeks of age, whereas CCR2^+^ peripheral immunocytes rarely infiltrated at 20 weeks of age. Infiltration of CX3CR1^+^ cells along the intramedullary lower motoneuron axonal tracts was prominent at 16 weeks of age (arrow). (**b**) Low magnification fluorescence images of *CCR2*^RFP/WT^/*CX3CR1*^GFP/WT^ (non-mSOD1 type) and *SOD1*^G93A^/*CCR2*^RFP/WT^/*CX3CR1*^GFP/WT^ (mSOD1-Tg) mouse sciatic nerves. In the sciatic nerves of a mSOD1-Tg mouse, CX3CR1^+^ cell infiltrations were visible as early as 4 weeks of age and CX3CR1^+^CCR2^+^ cells appeared at 8 weeks of age (pre-symptomatic stage). These infiltrated cells showed a foamy appearance (inset in the 8 weeks merged image). Such inflammatory infiltrates were not seen in non-mSOD1 type mice. (**c**) The quantitative analysis of CCR2^+^ CX3CR1^−^ (red), CCR2^−^CX3CR1^+^ (green), and CX3CR1^+^CCR2^+^ (yellow) areas (%) in sciatic nerves. In *SOD1*^G93A^ mice, numbers of all types of macrophages (CCR2^−^CX3CR1^+^ green, and CCR2^+^CX3CR1^+^ yellow macrophages) increased as the disease progressed, whereas CCR2^+^CX3CR1^−^ red immune cells did not. Scale bars: (**a**) 500 μm; (**b**) 50 μm and 10 μm (inset). n.s. = not significant. *p < 0.05; **p < 0.001; ***p < 0.0001.

Microscopic analysis of CX3CR1 and CCR2 in the spinal cord from *SOD1*^G93A^/*CCR2*^RFP/WT^/*CX3CR1*^GFP/WT^ mice revealed that the accumulation of CX3CR1^+^ microglia occurred mainly in the lumbar anterior horns at 12 weeks of age (at the time of clinical disease onset) without the infiltration of CCR2^+^ peripheral immunocytes (Fig. 2a). At 16 weeks of age (at the progressive stage), microglial activation became more robust, particularly along the intramedullary ventral roots, whereas CCR2^+^ cells were rarely observed. At 20 weeks of age (the moribund stage), CX3CR1^+^ activated microglia/macrophages without a foamy shape were present throughout the entire spinal cord section, whereas fewer CCR2^+^ cells had infiltrated the lesion (Fig. 2a).

### CX3CR1^+^ and CCR2^+^ cells infiltrate peripheral nerves earlier than in the spinal cord

Because a previous study reported the earlier deposition of mSOD1 protein in the lumbar spinal cord, sciatic nerves, and gastrocnemius muscles compared with the cervical spinal cord^22^, we immunohistochemically compared monocyte/macrophage infiltration into the sciatic nerves of *SOD1*^G93A^/*CCR2*^RFP/WT^/*CX3CR1*^GFP/WT^ and *CCR2*^RFP/WT^/*CX3CR1*^GFP/WT^ mice (non-mSOD1 type littermate). In the sciatic nerve of *CCR2*^RFP/WT^/*CX3CR1*^GFP/WT^ mice, CX3CR1^+^ resident macrophages were evenly scattered, whereas CCR2^+^ immunocytes were rarely seen at 16 weeks of age (Fig. 2b). We did not observe CX3CR1^+^CCR2^+^ cells in the sciatic nerves of non-mSOD1 type mice. Conversely, the sciatic nerves of *SOD1*^G93A^/*CCR2*^RFP/WT^/*CX3CR1*^GFP/WT^ mice showed a marked increase of CX3CR1^+^CCR2^−^ (green) cells, but not CX3CR1^−^CCR2^+^ (red) or CX3CR1^+^CCR2^+^ (yellow) cells at 4 weeks of age. At 8 weeks of age (pre-onset stage), CX3CR1^+^CCR2^+^ (yellow) cells appeared in the sciatic nerve, and successively higher numbers appeared at 12 to 16 weeks of age (onset to progressive phases) (Fig. 2b, c). The increase ratio was more prominent for green cells compared with yellow cells. Many green cells had a foamy appearance (Fig. 2b inset).

### CCR2 deficiency aggravates the clinical course of SOD1^G93A^ ALS mice

**Figure 3:**
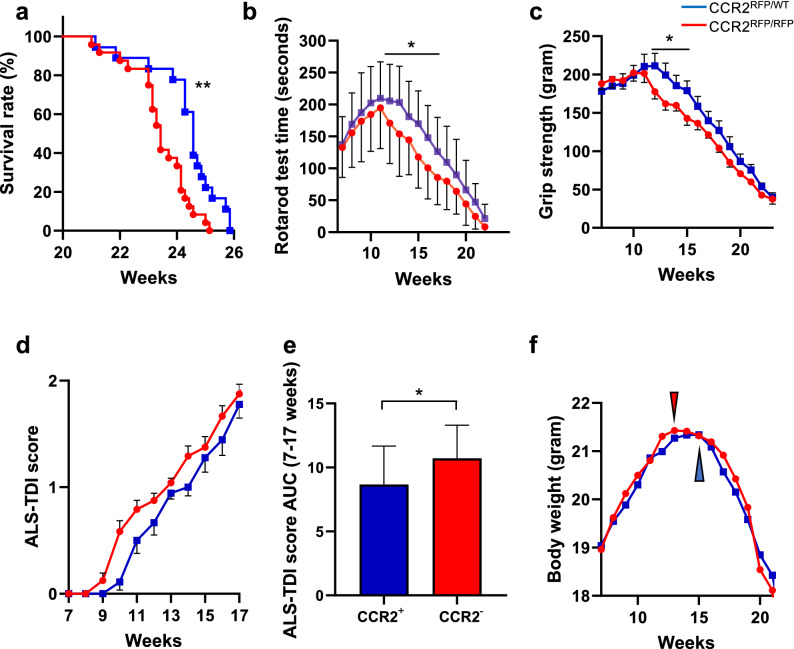
Clinical manifestations of CCR2-positive and CCR2-deficient *SOD1*^G93A^ mice. (**a**) Median survival time, (**b**) rotarod test, (**c**) grip strength, (**d**) ALS-TDI score, and (**e**) area under the curve (AUC) of ALS-TDI scores measured from 7 to 17 weeks of age in (**d**). (**f**) Mean age of disease onset was defined as when mice reached peak body weight (CCR2-positive *SOD1*^G93A^ mice, n = 24, and CCR2-deficient *SOD1*^G93A^ mice, n = 18). The log-rank test was used to compare median survival time and onset time by body weight. Student’s *t* test was used for statistical comparisons of rotarod test and grip strength. The Mann–Whitney *U*-test was used for statistical comparisons of the AUC of the ALS-TDI scores. *p < 0.05; **p < 0.001.

To clarify the effects of CCR2 deletion upon mSOD1-ALS, *SOD1*^G93A^/*CCR2*^RFP/WT^ mice (CCR2-positive *SOD1*^G93A^ mice, n = 24) and *SOD1*^G93A^/*CCR2*^RFP/RFP^ mice (CCR2-deficient *SOD1*^G93A^ mice, n = 18) were compared by measuring body weights, rotarod test, grip strength, and ALS-Therapy Development Institute (TDI) scores. CCR2-deficient *SOD1*^G93A^ mice showed a 6-day acceleration in progression to the moribund stage (164.0 ± 1.48 days vs. 170.4 ± 2.06 days, *p* = 0.0047; Fig. 3a) compared with CCR2-positive *SOD1*^G93A^ mice. Clinical signs were also significantly exacerbated in CCR2-deficient mice as determined by the rotarod test (*p* < 0.05; 12 weeks to 17 weeks; Fig. 3b), grip strength (*p* < 0.05; 13 weeks to 15 weeks; Fig. 3c), and ALS-TDI scores (area under the curve from 7 to 19 weeks of age: *p* = 0.031; Fig. 3d, e) compared with CCR2-positive *SOD1*^G93A^ littermates. The peak body weight was reached earlier in CCR2-deficient *SOD1*^G93A^ mice than in CCR2-positive *SOD1*^G93A^ littermates, although the difference was not statistically significant (13.83 ± 0.34 weeks vs. 14.83 ± 0.35 weeks, respectively, *p* = 0.188; Fig. 3f).

### CCR2 deficiency accelerates mSOD1 accumulation in peripheral nerves

**Figure 4:**
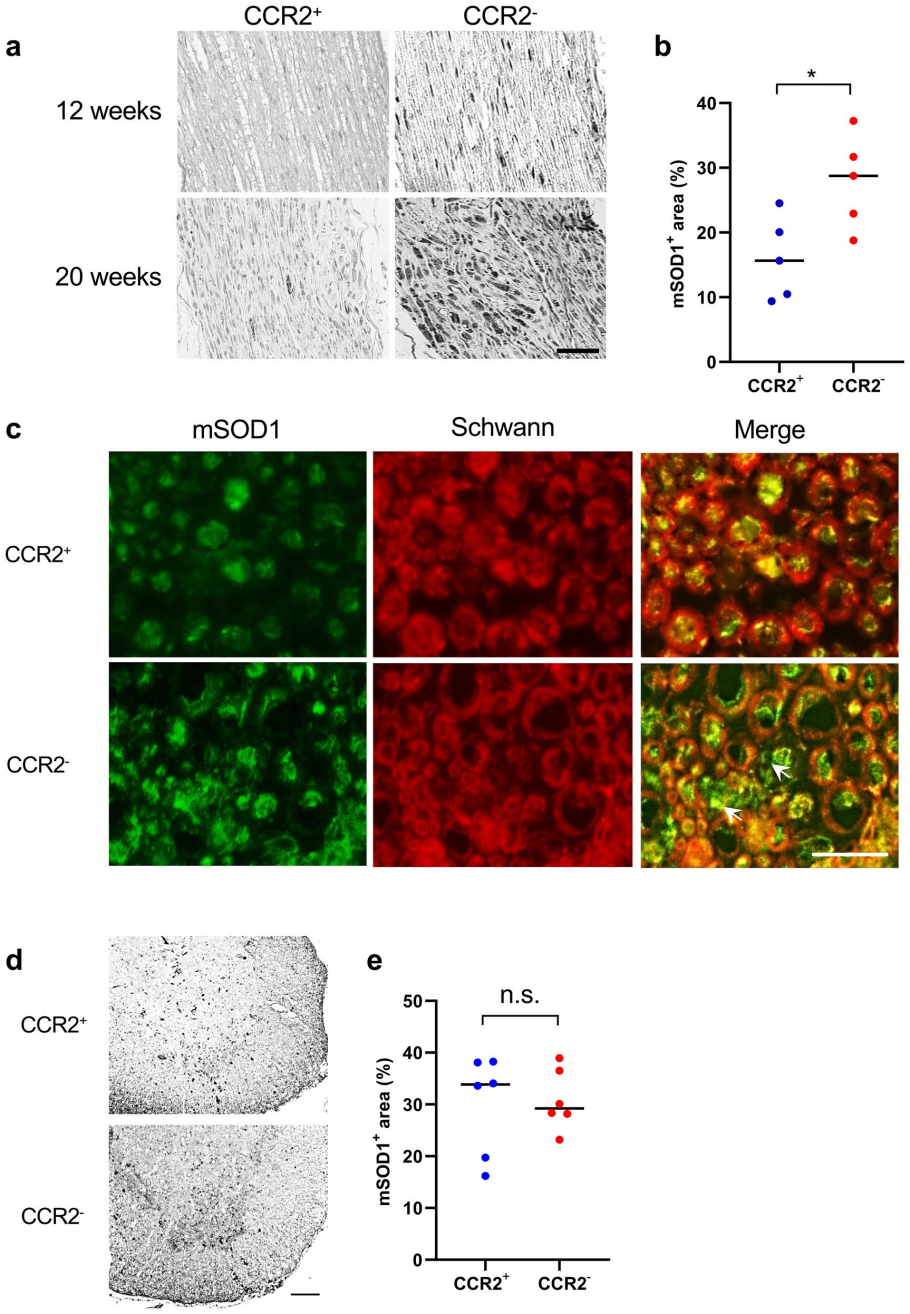
Aggravation of mSOD1 accumulation in the nerve by CCR2 ablation. (**a**) Immunostaining for mSOD1 in the sciatic nerves of CCR2-deficient *SOD1*^G93A^ mice and CCR2-positive littermates. (**b**) Accumulation of mSOD1 protein was significantly increased in CCR2-deficient *SOD1*^G93A^ mice than in CCR2-positive *SOD1*^G93A^ mice at 12 weeks of age (*p* = 0.032, n = 5). (**c**) Double immunostaining for mSOD1 and Schwann cells indicated a greater accumulation of mSOD1 protein in the sciatic nerves of CCR2-deficient *SOD1*^G93A^ mice showing an overflow of mSOD1 protein from Schwann cells and in the myelin sheath (arrows) compared with CCR2-positive *SOD1*^G93A^ mice at 12 weeks of age. (**d**) Immunostaining for mSOD1 protein in the lumbar spinal cord of CCR2-deficient *SOD1*^G93A^ mice and CCR2-positive littermates at 12 weeks of age. (**e**) There was no difference in the mSOD1^+^ area (%) between the two mouse genotypes at 12 weeks of age (*p* = 0.85, n = 6). The unpaired *t* test was used for statistical comparisons. Scale bars: (**a**) 20 μm; (**c**) 10 μm; (**d**) 100 μm. *p < 0.05. n.s. = not significant.

Immunohistochemical analysis with anti-human specific mSOD1 antibody revealed a significant increase in mSOD1 protein aggregation in the sciatic nerves of CCR2-deficient *SOD1*^G93A^ mice compared with CCR2-positive littermates (*p* = 0.032; Fig. 4a, b) at 12 weeks of age (onset period). The accumulation of mSOD1 protein was also confirmed by western blotting (Supplementary Fig. 1). In the sciatic nerves, mSOD1 protein immunostaining was surrounded by the myelin sheath in CCR2-positive *SOD1*^G93A^ mice. Conversely, CCR2-deficient *SOD1*^G93A^ mice demonstrated immunostaining of mSOD1 protein aggregation inside and outside the myelin sheath (Fig. 4c), which suggests that excessive mSOD1 protein accumulation had overflowed into the myelin sheath (arrows in Fig. 4c). Conversely, there were no significant differences in mSOD1 protein aggregation in the lumbar spinal cord of CCR2-deficient *SOD1*^G93A^ mice and CCR2-positive littermates (*p* = 0.77; Fig. 4d, e) at 12 weeks of age (onset period).

### CCR2 deficiency worsens anterior horn cell loss and axonal deformation

**Figure 5:**
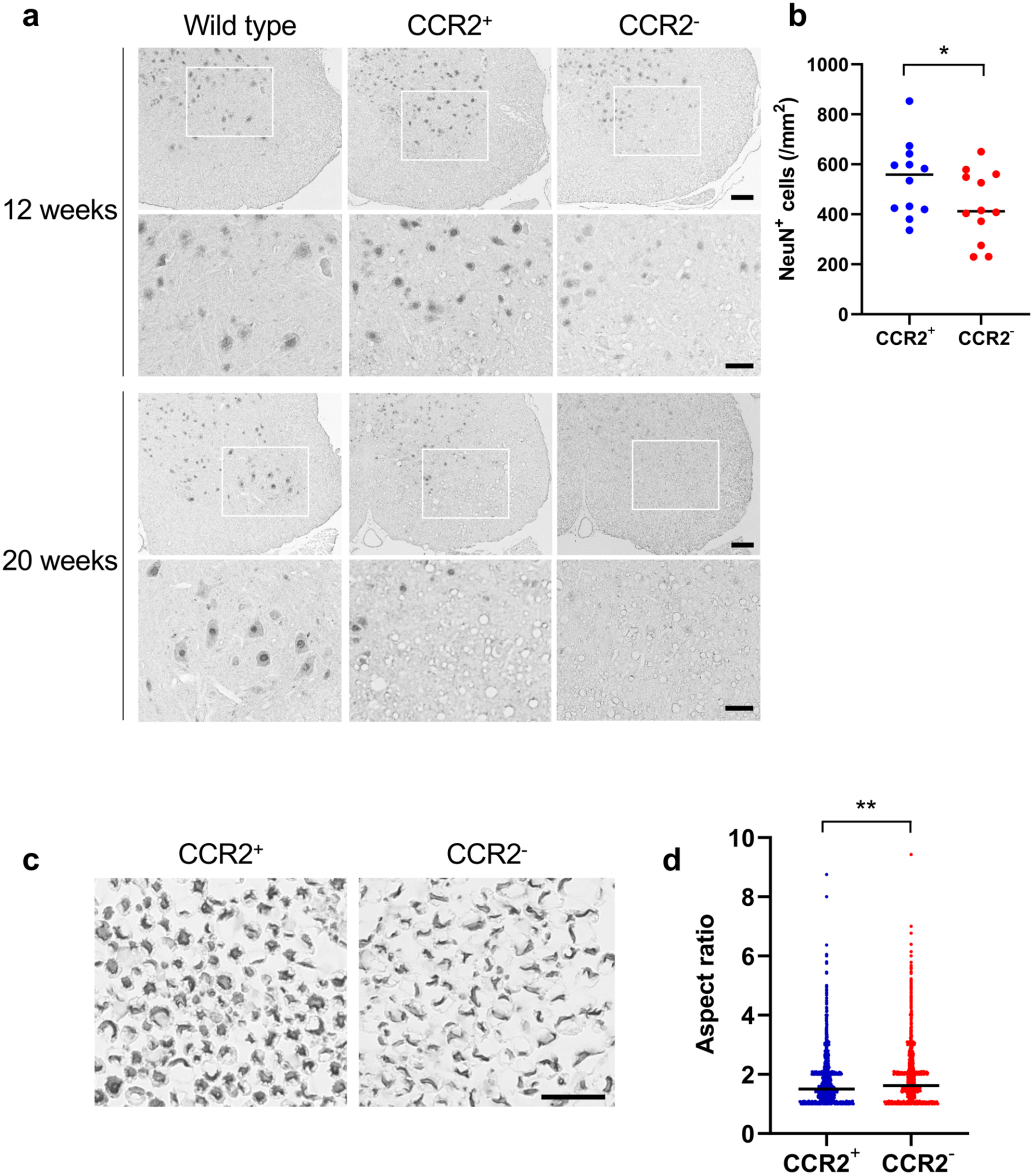
Facilitation of neuro-axonal degeneration by CCR2 ablation. (**a**) NeuN immunostaining revealed increased anterior horn neuronal cell loss in CCR2-deficient *SOD1*^G93A^ mice compared with CCR2-positive *SOD1*^G93A^ mice at 12 and 20 weeks of age. Inset areas in the low magnified images are enlarged under each figure. (**b**) CCR2-deficient *SOD1*^G93A^ mice showed a significant decrease in NeuN^+^ cell numbers compared with CCR2-positive *SOD1*^G93A^ mice at 12 weeks of age (*p* = 0.033, n = 12). (**c**) SMI 31/32 immunostaining of sciatic nerve transverse sections indicated axons in CCR2-deficient *SOD1*^G93A^ mice had crescent-shaped deformation compared with CCR2-positive *SOD1*^G93A^ littermates at 8 weeks of age. (**d**) The aspect ratio (the ratio of the major to the minor axis length) of CCR2-deficient *SOD1*^G93A^ mouse axons was significantly larger than that of CCR2-positive *SOD1*^G93A^ mouse axons at 8 weeks of age (*p* < 0.001, n = 5). Scale bars: (**a**) 100 μm and 50 μm; c, 20 μm. The horizontal line represents the mean value in (**b**, **d**). *p < 0.05; **p < 0.001.

**Figure 6:**
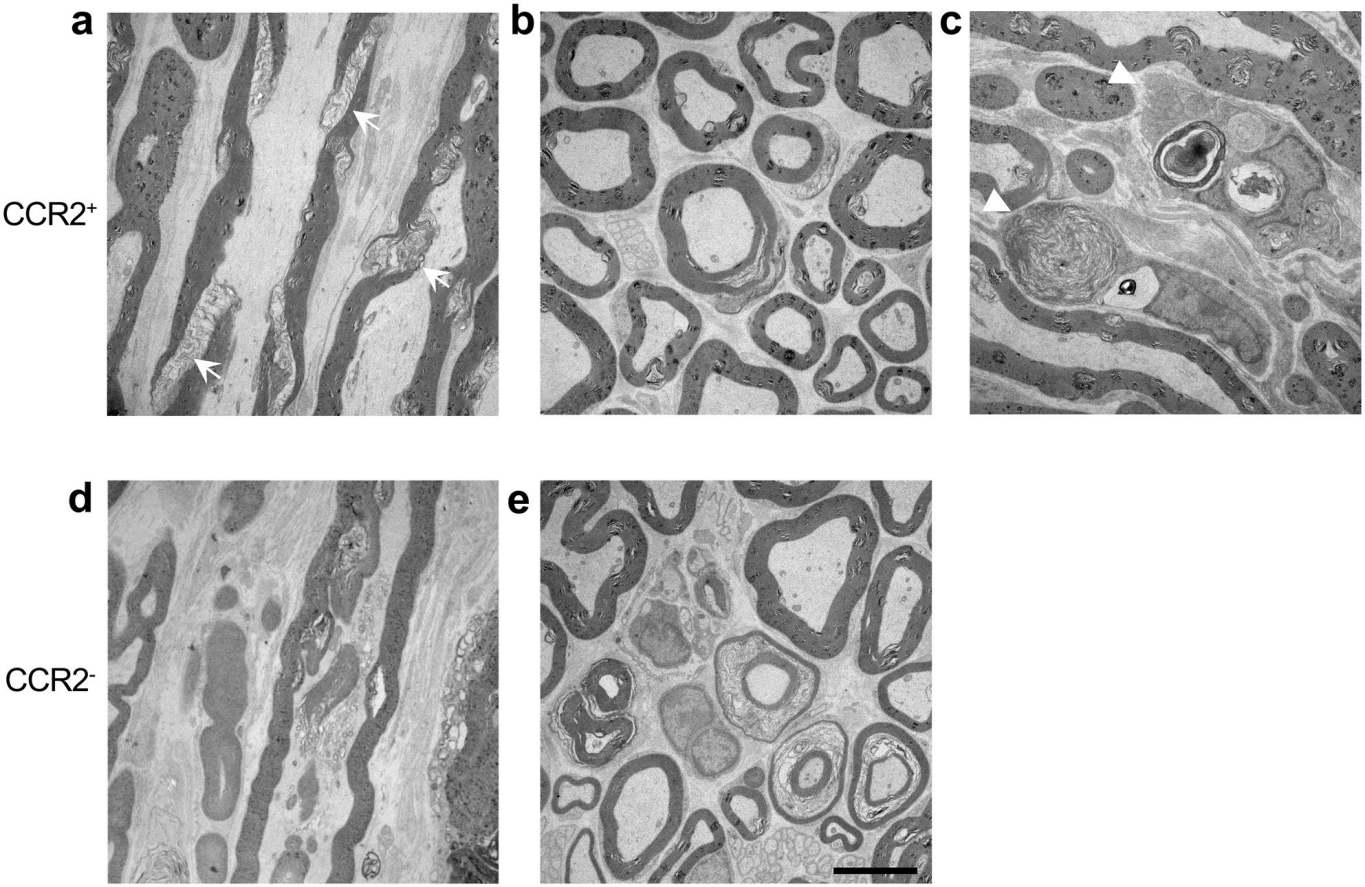
Electron microscopic findings of the sciatic nerve. (**a**) The longitudinal section of the sciatic nerve from a CCR2-positive *SOD1*^G93A^ mouse at 12 weeks of age shows preserved axonal structure and some derangement of the Schmidt-Lanterman incisures (arrow). (**b**) Cross-section of the sciatic nerve from a CCR2-positive *SOD1*^G93A^ mouse at 20 weeks of age with relatively preserved axons and some vesiculation of the myelin sheath. (**c**) Foamy macrophages containing cell and myelin debris are visible adjacent to the axons in the longitudinal section of the sciatic nerve from a CCR2-positive *SOD1*^G93A^ mouse at 12 weeks of age (arrowhead). (**d**) Longitudinal section of the sciatic nerve from a CCR2-deficient *SOD1*^G93A^ mouse at 12 weeks of age with disruption of intra-axonal structures. (**e**) Cross-section of the sciatic nerve from a CCR2-deficient *SOD1*^G93A^ mouse at 20 weeks of age with many disrupted, shrunken axons and marked vesiculation of the myelin sheath. Scale bar: 5 μm.

The loss of anterior horn cells as evaluated by NeuN immunostaining was facilitated in CCR2-deficient *SOD1*^G93A^ mice compared with CCR2-positive *SOD1*^G93A^ mice (Fig. 5a). Significantly fewer anterior horn cell numbers were observed in CCR2-deficient *SOD1*^G93A^ mice than in CCR2-positive littermates at 12 weeks of age (*p* = 0.033) (Fig. 5b). Moreover, SMI 31/32 immunostaining of peripheral nerve transverse sections revealed that the axons of CCR2-deficient *SOD1*^G93A^ mice, but not CCR2-positive *SOD1*^G93A^ mice, already showed crescent-like deformation at 8 weeks of age (pre-onset period) (Fig. 5c). The aspect ratio (calculated as the ratio between major and minor axis lengths) was significantly higher in CCR2-deficient *SOD1*^G93A^ mice than in CCR2-positive *SOD1*^G93A^ mice (aspect ratio: 1.779 vs. 1.683, *p* = 4.11 × 10^−16^; Fig. 5d), which indicates accelerated axonal deformation in CCR2-deficient *SOD1*^G93A^ mouse peripheral nerves. Electron microscopy also confirmed that axons were more deranged in CCR2-deficient *SOD1*^G93A^ mice compared with CCR2-positive *SOD1*^G93A^ mice, whereas foamy macrophages filled with myelin and other cell debris were more frequently observed in CCR2-positive *SOD1*^G93A^ mice compared with CCR2-deficient *SOD1*^G93A^ mice (Fig. 6).

### CCR2 deficiency diminishes anti-inflammatory M2 macrophage infiltration into the peripheral nerves

**Figure 7:**
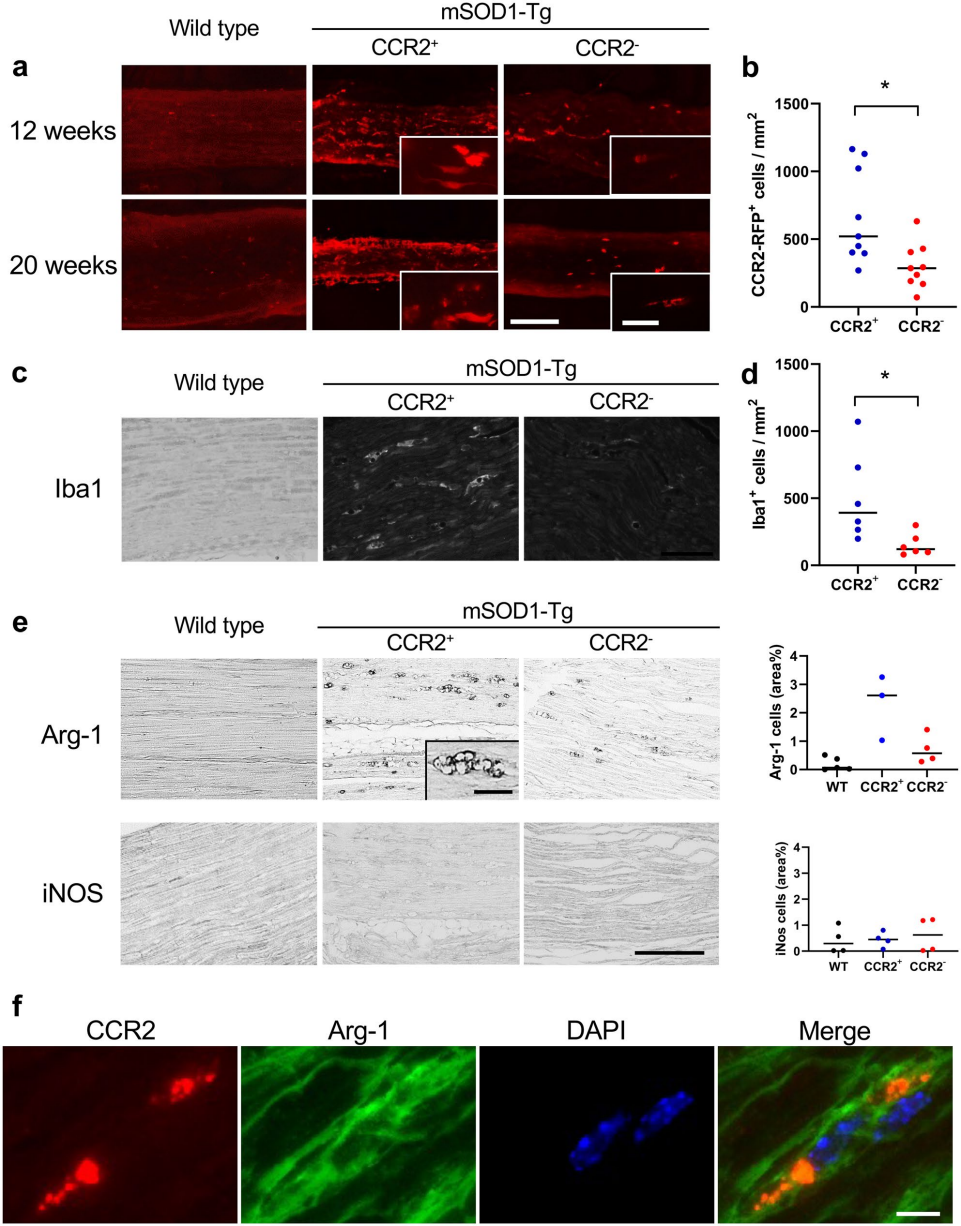
Inhibition of macrophage infiltration into the sciatic nerve by CCR2 ablation. (**a**) Fluorescent microscopic analysis of unstained sciatic nerve tissues of CCR2-positive non-mSOD1 type mice, CCR2-positive *SOD1*^G93A^ mice, and CCR2-deficient *SOD1*^G93A^ mice. The influx of CCR2-RFP^+^ cells into the sciatic nerve was markedly diminished in CCR2-deficient *SOD1*^G93A^ mice than in CCR2-positive littermates. (**b**) The number of CCR2-RFP^+^ cells was significantly reduced in CCR2-deficient *SOD1*^G93A^ mice compared with CCR2-positive littermates (*p* = 0.021, n = 9). (**c**) Immunostaining for Iba1 in sciatic nerves shows a marked reduction of CCR2^+^ cell infiltrates in CCR2-deficient *SOD1*^G93A^ mice compared with CCR2-positive littermates. (**d**) The number of Iba-1^+^ cells was significantly reduced in CCR2-deficient *SOD1*^G93A^ mice compared with CCR2-positive littermates (*p* = 0.047, n = 6). (**e**) Immunostaining for arginase-1 (Arg-1), an M2 marker, and inducible nitric oxide synthase (iNOS), an M1 marker, of the sciatic nerve, demonstrated infiltrated macrophages were Arg-1^+^ but iNOS^-^, which suggests an M2-deviated phenotype. Inset in (**e**) indicates the foamy appearance of Arg-1^+^ cells. (**f**) Foamy macrophages with Arg-1 are CCR2-positive. Scale bars: (**a**) 100 μm and 20 μm (inset); (**c**) 50 μm; (**e**) 50 μm; (**f**) 20 μm. The horizontal line in (**b**, **d**) represents the mean value. The unpaired *t *test was used for statistical comparisons. *p < 0.05.

As shown in Fig. 7a, CCR2^+^ cell infiltration into the sciatic nerve was markedly diminished in CCR2-deficient *SOD1*^G93A^ mice compared with CCR2-positive littermates at 12 (onset stage) and 20 weeks of age (moribund stage) (Fig. 7a). As a result, there were significantly fewer CCR2-RFP positive cells in the sciatic nerves of CCR2-deficient mice compared with CCR2-positive *SOD1*^G93A^ mice at 12 weeks of age (*p* = 0.021; Fig. 7b). In addition, Iba1 immunostaining also indicated a significant decrease in Iba1^+^ macrophages in the sciatic nerves of CCR2-deficient *SOD1*^G93A^ mice compared with CCR2-positive *SOD1*^G93A^ mice at 12 weeks of age (*p* = 0.047; Fig. 7c, d). To further characterize the phenotype of infiltrating macrophages in the peripheral nerves of mSOD1 ALS mice, immunostaining for arginase-1 (Arg-1), a marker of anti-inflammatory M2 macrophages, and inducible nitric oxide synthase (iNOS), a marker of pro-inflammatory M1 macrophages, was performed. In the sciatic nerves of both mouse genotypes, infiltrated foamy macrophages were immunopositive for Arg-1 but negative for iNOS (Fig. 7e). CCR2 positive foamy-macrophages that were immunopositive for CD68 contained Arg-1 (Fig. 7f; Supplementary Fig. 2). Regarding T cell infiltration into the peripheral nerves, histological analysis indicated CD3^+^ T cells were rarely detected in the sciatic nerves of CCR2^+^ or CCR2^−^ *SOD1*^G93A^ mice and that there was no significant difference in cell numbers between these mouse strains (Supplementary Fig. 3).

### Differentiated macrophages are decreased in the sciatic nerves of CCR2-deficient mice

Flow cytometric analysis of cells isolated from sciatic nerves revealed comparable leukocyte ratios between CCR2^+^ and CCR2^−^ mSOD1-Tg mice, except for a decrease in CD11b^+^/CD11c^+^ macrophages in CCR2-deficient mice (CD11b^+^/CD11c^+^ cell population ratio in CD45^+^ gate (%) (CCR2^+^mSOD1 vs. CCR2^-^mSOD1, mean ± SEM = 25.40 ± 2.577 vs. 12.67 ± 1.619, *p* = 0.0111). There were no significant differences in the CD45^+^CD3^+^ T cell population ratio in the sciatic nerves of CCR2^+^ and CCR2^−^ mSOD1-Tg mice (Supplementary Fig. 4).

### CCL2 production by Schwann cells is unchanged by CCR2 deficiency

**Figure 8:**
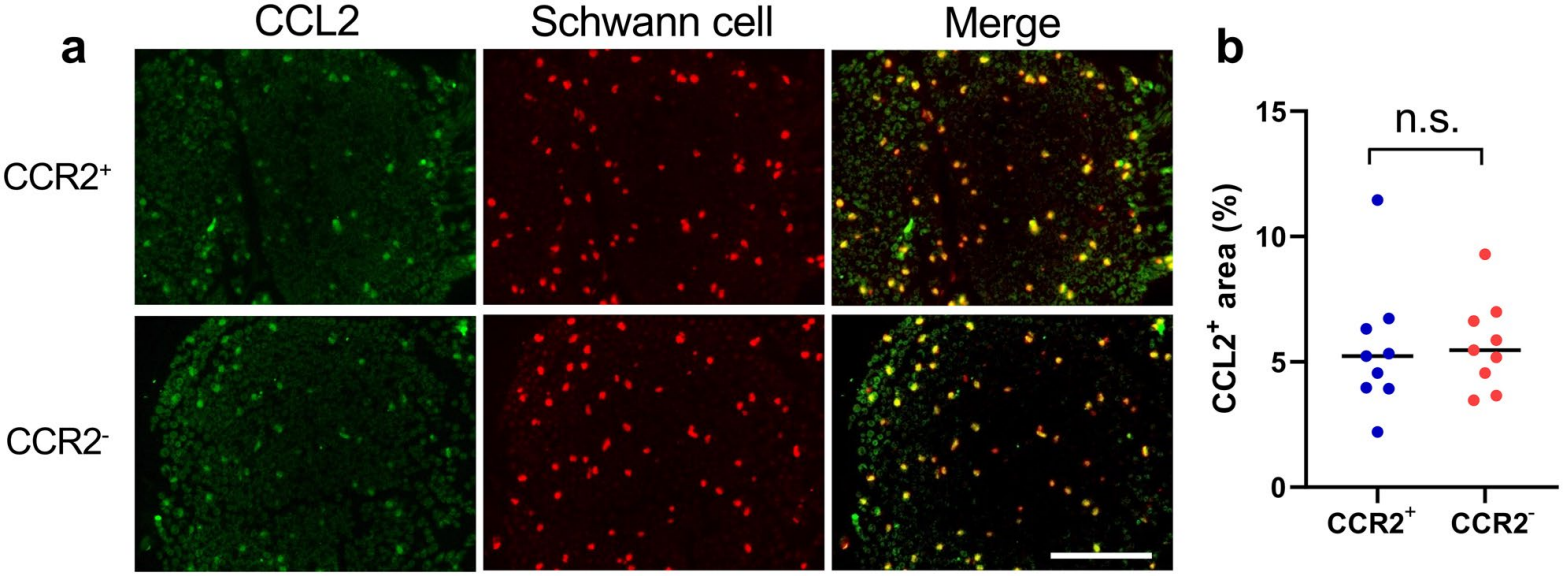
CCL2 production by Schwann cells. (**a**) Double immunostaining of CCR2-deficient and CCR2-positive *SOD1*^G93A^ mouse sciatic nerves at 16 weeks with anti-Schwann cell and anti-CCL2 antibodies. The merged images of both antibodies indicate the production of CCL2 by Schwann cells in both mouse genotypes. Scale bar: 20 μm. (**b**) The CCL2^+^ area (%) was not significantly different between CCR2-deficient and CCR2-positive *SOD1*^G93A^ mice. The horizontal line represents the mean value. The unpaired *t* test was used for statistical comparisons (*p* = 0.92, n = 9). n.s. = not significant.

Double immunostaining with anti-Schwann cell antibody (human peripheral nerve extract antigen, mouse IgM antibody)^23^ and anti-CCL2 antibody demonstrated that CCL2 was mainly co-localized with Schwann cells in the sciatic nerves of *SOD1*^G93A^ mice at 16 weeks, regardless of the presence or absence of CCR2 (Fig. 8a). This indicated that Schwann cells are the primary source of CCL2 in peripheral nerves. The numbers of CCL2^+^ cells in the sciatic nerve were not significantly different between CCR2-deficient and CCR2-positive *SOD1*^G93A^ mice (*p* = 0.92; Fig. 8b). The anti-Schwann cell antibody we used in this study was a mouse IgM antibody that uses human peripheral nerve extract as its antigen.

### Phagocytic activity of isolated macrophages is unaltered by CCR2 deficiency

Finally, we measured the phagocytic activity of macrophages isolated from *CCR2*^RFP/RFP^ *SOD1*^G93A^ mice, *CCR2*^RFP/WT^ *SOD1*^G93A^ mice, *CCR2*^RFP/RFP^ mice, and *CCR2*^RFP/WT^ mice. There was no difference in phagocytic activity among these mice in vitro (Supplementary Fig. 5), which suggests that a deficiency of CCR2 or the presence of mSOD1 does not influence the phagocytic activity of isolated macrophages.

## Discussion

The role of peripheral blood-borne macrophages bearing CCR2 recruited into the neural tissues has long been a critical question in ALS. To address this issue, we studied mSOD1 ALS mice with genetically labeled macrophages or with CCR2 deficiency. We found that before the onset of clinical symptoms, CCR2^+^ macrophages that phagocytized mSOD1 protein had already infiltrated the peripheral nerves much earlier than into the spinal cord. Furthermore, CCR2 ablation clinically accelerated the disease progression and worsened the pathology, as determined by NeuN^+^ neuronal loss in the spinal anterior horns and axonal derangement in the peripheral nerves. CCR2 ablation also markedly increased the accumulation of mSOD1 protein in the peripheral nerves and, to a lesser extent, in the spinal cord compared with CCR2-positive mice. Flow cytometric analysis revealed a comparable number of CD3^+^ T cells were detected in the sciatic nerves of 8-week-old mSOD1-Tg mice with and without CCR2. However, we also detected the suppressed infiltration of the CD11b^+^/CD11c^+^ cell population, which indicates that activated macrophages derived from monocytes^24^ were decreased in the sciatic nerves of CCR2-deficient mice. A decreased infiltration of CCR2^+^ macrophages, which phagocytized mSOD1 protein and expressed Arg-1, an M2 marker, but not iNOS, an M1 marker, in the peripheral nerves was also observed. These findings suggest that CCR2^+^ macrophages recruited into the peripheral nerves from the blood exert neuroprotective functions on the lower motor neurons in mSOD1 ALS and that the clearance of abnormal mSOD1 protein from peripheral nerves by these cells is a hitherto underestimated host protective mechanism (Supplementary Fig. 6).

The *SOD1*^G93A^/*CCR2*^RFP/WT^/*CX3CR1*^GFP/WT^ mice started to deteriorate at 12 weeks of age and died at 21 weeks of age, which is similar to the reported clinical course of mSOD1^G93A^ ALS mice^22^. In the *SOD1*^G93A^/*CCR2*^RFP/WT^/*CX3CR1*^GFP/WT^ mice, we observed that mSOD1 accumulated in the peripheral nerves much earlier (4 weeks of age) than in the spinal cord (12 weeks of age), which is in accord with a previous report describing the earlier accumulation of mSOD1 protein in the sciatic nerve compared with the lumbar spinal cord (2.6-fold increase in the sciatic nerve vs. 1.8-fold increase in the lumbar cord at 30 days of age compared with at birth, and remaining consistently higher in the sciatic nerve than in the lumbar cord until 120 days of age)^22^. These observations support distal axonopathy as a primary mechanism of the lower motor neuron death in mSOD1 ALS^6,25^. We also confirmed the accumulation of mSOD1 in the DRG and, to a lesser extent, in the dorsal roots as previously reported, which explains the sensory system involvement observed in mSOD1 ALS patients and model animals^26^. Because DRGs are bipolar cells, we hypothesized that mutant protein transported by an afferent mechanism is not cleared in CNS regions where there is no or little peripheral macrophage infiltration, unlike the sciatic nerve, which harbors high numbers of peripheral macrophages. It was suggested that there may be differences in the characteristics of axonal transport between motor and sensory neurons in the SOD1 mouse model related to differences in dynein and dynactin functions^27^.

In line with the earlier accumulation of mSOD1 protein in the peripheral nerves, we found increased CX3CR1^+^ macrophages infiltration into the sciatic nerve as early as 4 weeks of age and the subsequent infiltration of CX3CR1^+^CCR2^+^ macrophages at 8 weeks of age. CX3CR1^+^ macrophages, which were observed at 4 weeks of age, are considered resident macrophages, whereas CX3CR1^+^CCR2^+^ macrophages are thought to be peripheral blood-borne macrophages. CCR2 single positive cells are considered peripheral blood-derived immune cells, other than monocytes/macrophages. We also found a rapid increase in CX3CR1^+^CCR2^−^ green cell numbers in peripheral nerves in the progressive phase of the disease. Also, the appearance of green cells was different between 4 weeks (thin shape) and 8 weeks (foamy shape). Figure 2c shows an increase of CX3CR1^+^CCR2^+^ yellow cells. RFP expression was reduced after monocytes infiltrated tissues because its half-life is up to 4.6 days^28^. As shown in Supplementary Fig. 7, peripheral yellow monocytes infiltrate the sciatic nerve and then differentiate into macrophages, which lose their red color, which results in green foamy cells. The increase of yellow cells at 12 and 16 weeks indicates the acceleration of monocyte infiltration into lesions. We assume that the green foamy cells were initially infiltrated as blood-borne monocytes with yellow color, but soon after the CCR2 expression was down-regulated along with RFP, which resulted in green foamy cells. These findings are in line with previous reports that CCR2 on activated monocytes is rapidly internalized, and that the synthesis of CCR2 is down-regulated^15^. Our results are compatible with previous results showing CD68^+^ macrophages infiltrated the ventral roots of mSOD1^G93A^ mice at 60 days of age^22^. Although it was unclear whether these cells were protective or worsened disease progression, we hypothesize these macrophages scavenge mSOD1 protein to protect peripheral nerves. This notion is supported by the observations that these macrophages were full of mSOD1 protein and that a deficiency of these cells by CCR2 ablation markedly facilitated mSOD1 accumulation in the nerves and promoted the death of the lower motor neurons. Flow cytometric analysis revealed a decrease of the CD11b^+^/CD11c^+^ cell population in CCR2 deficient mice. Because CCL2 induces the chemotaxis of CCR2 positive cells and induces the differentiation of monocytes, this disparity in the CD11b^+^/CD11c^+^ cell ratio might result from the lack of CCL2 signaling in CCR2 deficient monocytes. Activated macrophages are subcategorized into several phenotypes, including M1 pro-inflammatory and M2 anti-inflammatory. The direction of activation induced by CCL2 depends on the inflammatory circumstances^29,30^. We confirmed that the foamy macrophages were all Arg-1 positive M2 phenotype, which indicated that the lack of CCL2 signaling in *CCR2*^RFP/RFP^ *SOD1*^G93A^ mice decreased the infiltration of CCR2-positive cells into the sciatic nerve and inhibited macrophage differentiation into M2 macrophages.

We confirmed that CCL2 production by Schwann cells, which are the primary source of CCL2 in nerves^31,32^, was unaffected in *CCR2*^RFP/RFP^ *SOD1*^G93A^ mice. Thus, our findings are in accord with previous reports showing that CCL2 expression in the sciatic nerve was not altered by *CCR2* gene deletion^33^ and that in peripheral nerve injury, CCR2-deficient mice had decreased macrophage accumulation in the sciatic nerve and DRG^34,35^. Whether CCR2 deficiency reduces phagocytic capacity is controversial^36,37^; however, we confirmed that the phagocytic activity of the isolated macrophages was unaffected by CCR2 ablation, at least in *SOD1*^G93A^/*CCR2*^RFP/RFP^ mice. Collectively, these findings indicate that the impaired migratory activity and the inhibition of M2-directed differentiation of macrophages related to the lack of CCL2-CCR2 signaling in the inflammatory milieu where abnormal proteins are accumulated contributed to the insufficient clearance of misfolded mSOD1 protein in peripheral nerves, which results in the accelerated loss of anterior horn cells and exacerbated disease in CCR2-deficient *SOD1*^G93A^ mice. In other neurodegenerative diseases, CCR2 deficiency was also reported to accelerate disease^38,39^. Notably, CCR2 deficiency particularly aggravated amyloid β clearance in an Alzheimer’s disease animal model, thereby inducing accelerated deterioration^38,39^. Taken together, these findings suggest CCR2^+^ peripheral blood-borne macrophages clear abnormal proteins, at least in the early stage of the disease, contributing to disease protection in neurodegenerative disorders, such as ALS, caused by abnormal protein accumulation.

CCR2 is abundantly expressed in M1 pro-inflammatory macrophages. Thus, CCR2 ablation is likely to inhibit M1 macrophage mobilization from peripheral blood to nerves. Given the pro-inflammatory properties of M1 macrophages, the neuroprotective action of peripheral blood-borne CCR2^+^ M1 macrophages in mSOD1 ALS is unexpected. However, importantly, these CCR2^+^ macrophages co-expressed CX3CR1 in the peripheral nerves of mSOD1 ALS mice, which suggests an intermediate phenotype, transitioning from pro-inflammatory M1 to anti-inflammatory M2 macrophages after invasion into the peripheral nerve. Macrophages were reported to switch phenotypes according to their microenvironment^7,40^. In peripheral nerve and spinal cord injury models, a transition from M1 to M2 phenotype was reported, and such intermediate macrophages had anti-inflammatory and neuroprotective properties^41^. Therefore, CCR2^+^ peripheral blood-borne macrophages that phagocytose mSOD1 may also be neuroprotective for peripheral nerves, thereby extending lower motor neuron survival time in mSOD1 ALS mice. There are several possibilities why the pathologic process still progresses even in the presence of macrophage clearance of misfolded mSOD1. Upper motor neurons are behind the blood–brain barrier, which may disturb macrophage clearance mechanisms in the early stage. Our results showed that CCR2^+^ cell infiltration into the spinal cord was only observed in the end-stage. Furthermore, there may be other neuropathic pathways in which the macrophage clearance mechanism does not work. The clinical course of CCR2^+^ mSOD1 mice eventually catches up at later periods. We hypothesized that this was related to the accumulation of mSOD1 protein in upper motor neurons because macrophage infiltration is blocked by the blood–brain barrier, and thus mSOD1 protein in the upper motor neuron cannot be removed.

Our study had several limitations. First, because of the small amount of CCR2-single positive red cells (T, B, and dendritic cells) in the sciatic nerve, we did not examine CCR2^+^ leukocyte involvement. It was reported that *SOD1*^*G93A*^*PU.*^*−/−*^ mice lacking CD3^+^ T cells had shorter life spans after transplantation with CCR2-deficient bone marrow compared with wild-type bone marrow transplantation^42^. CCR2^+^ T cells were thought to facilitate glial neuroprotection in this model. In our model, a detailed time-course study of T cell infiltration into the spinal cord and peripheral nerves is required in the future. Second, the characterization of infiltrated CCR2^+^ macrophages and their transition to the M2 phenotype was performed by immunohistochemistry because relatively small numbers of macrophages were present in the nerves. Future studies should characterize macrophages isolated from peripheral nerves by microarray or single-cell RNA sequencing.

In summary, our study revealed an under-recognized mechanism of abnormal protein clearance by CCR2^+^ macrophages from the peripheral nerves of mSOD1 ALS mice, which is beneficial to the host. Because lower motor neuron axons are present in peripheral nerves, which are more accessible to peripheral blood macrophages than CNS tissues that are tightly surrounded by the blood–brain barrier, peripheral nerves might be a novel therapeutic target for the cell therapy of ALS by removing abnormal proteins and delivering neuroprotective factors.

## Materials and methods

### Mice and ethical statement

Transgenic mice for the human *SOD1*^G93A^ gene [B6SJL-TgN (SOD1*G93A) 1Gur/J; Stock Number: 002297] were purchased from the Jackson Laboratory (Bar Harbor, ME, USA). They were crossed with C57BL/6J mice (Clea Japan, Tokyo, Japan) to maintain the strains, and hemizygous animals were used for the experiments. Transgenic mice harboring the human *SOD1*^G93A^ gene were backcrossed to C57BL/6J mice for more than 15 generations. *CCR2*^RFP^ mice [B6.129 (Cg)-Ccr2^tm2.1Ifc^/J; Stock Number: 017586], and *CX3CR1*^GFP^ mice [B6.129P (Cg)-Ptprca Cx3cr1^tm1Litt^/LittJ; Stock Number: 008451] were also purchased from the Jackson Laboratory. Heterozygous *SOD1*^G93A^ mice and *CCR2*^RFP/RFP^ mice were crossed to obtain *SOD1*^G93A^/*CCR2*^RFP/WT^ mice, and then *SOD1*^G93A^/*CCR2*^RFP/WT^ mice and *CCR2*^RFP/RFP^ mice were crossed to obtain *SOD1*^G93A^/*CCR2*^RFP/WT^ mice and *SOD1*^G93A^/*CCR2*^RFP/RFP^ mice. In *CCR2*^RFP/RFP^ homozygotes, *CCR2* alleles are inactivated (CCR2-deficient phenotype) whereas CCR2-RFP labeling is preserved. In addition, to create *SOD1*^G93A^/*CCR2*^RFP/WT^/*CX3CR1*^GFP/WT^ mice, *SOD1*^G93A^/*CCR2*^RFP/WT^ mice and *CX3CR1*^GFP/GFP^ mice were crossed to obtain *SOD1*^G93A^/*CCR2*^RFP/WT^/*CX3CR1*^GFP/WT^ mice. This dual heterozygous mouse expressing both reporter proteins and their receptors at functional levels has been widely used to differentiate resident microglia from blood-derived monocytes/macrophages in various neurodegenerative models^15,43^. Non-*SOD1*^G93A^-transgenic littermates were used as a non-ALS model phenotype. All animals were maintained in an air-conditioned, specific-pathogen-free room with a time-controlled lighting system. The handling and sacrifice of all animals were conducted according to the guidelines for the proper conduct of animal experiments published by the Science Council of Japan and the ARRIVE (Animal Research: Reporting of In Vivo Experiments) guidelines for animal research. Ethical approval for the study was granted by the Animal Care and Use Committee of Kyushu University (#A30-051).

### Behavioral study

Body weights, performances in the rotarod test (ENV-576M; Neuroscience, Tokyo, Japan), grip strength (MK-380M, Muromachi-Kikai, Tokyo, Japan), and ALS-TDI neurological scoring^44^ were assessed once a week. For the assessment of motor function by rotarod, mice were habituated to stay on the stationary drum for 3 min before the training sessions. Habituation was repeated each time for 1 min just before the session. Mice were examined on the rotarod with an accelerating speed of 5 to 30 rpm over 300 s. The trials were performed three times, and the longest time was recorded. The time limit of each observation was 300 s. The time of disease onset was retrospectively determined as the time when the mice reached their peak body weight. For the assessment of grip strength, mice were lifted by the base of the tail and placed so that their front paws gripped the trapeze with their body horizontal. Each mouse was tested five times to obtain the best grip strength performance. The ALS-TDI neurological score was measured as follows: 0, normal gait is observed; 1, the hindlimb collapsed towards the lateral midline or trembled; 2, while walking, any part of the foot dragged along the cage bottom/table; 3, the hindlimb was not used for forwarding motion but was able to right itself within 10 s; and 4, rigid paralysis in the hindlimb and absence of righting reflex.

### Immunohistochemistry

All animals were deeply anesthetized with sevoflurane and perfused intracardially with saline followed by cold 4% paraformaldehyde (PFA) in phosphate-buffered saline (PBS). The lumbar cord, sciatic nerve, and ventral roots were subsequently removed, immersed for over 12 h in the same 4% PFA fixative at 4 °C, and processed for making paraffin-embedded materials or optimal cutting temperature compound-embedded frozen materials. Multiple 5-μm-thick paraffin-embedded sections and 10-μm-thick frozen sections were used for immunohistochemical staining. Paraffin-embedded sections were deparaffinized, and frozen sections were air-dried. Endogenous peroxidase activity was blocked by 3% H_2_O_2_ in methanol/PBS (1:1) for 10 min at room temperature. Sections were then incubated with primary antibodies at 4 °C overnight^45^. After rinsing, sections were subjected to either a streptavidin–biotin complex or an enhanced indirect immunoperoxidase method using Envision (Dako Cytomation, CA, USA). Immunoreactivity was detected using 3,3′-diaminobenzidine as the chromogen. The primary antibodies for mouse tissues are listed in Supplementary Table 1. For immunohistochemical staining, the sections were incubated with secondary antibodies conjugated to Alexa Fluor 488 or 594 (1:1000; Thermo Fisher, Rockford, IL, USA) and 4′,6-diamidino-2-phenylindole (DAPI) (Sigma-Aldrich, Tokyo, Japan) to stain cell nuclei, and mounted with Permafluor (#TA-030-FM; Thermo Scientific, Fremont, CA, USA). Tissues were observed with a fluorescence microscope (BZ-X700, Keyence, Tokyo, Japan).

### Western blotting

Sciatic nerves of mice were homogenized in 50 μl of lysis buffer containing 8% sodium dodecyl sulfate (SDS) and RIPA buffer (#16488-34; Nacalai Tesque, Kyoto, Japan). After centrifugation at 14,000×*g* for 10 min, the supernatants were collected. The protein concentration in the supernatant was measured using a DC protein assay kit (Bio-Rad, Tokyo, Japan). Total protein (6 μl each) was separated by SDS polyacrylamide gel electrophoresis (15%). Western blotting analysis was performed using anti-mSOD1 (500 ng/ml) according to a previously described method^46^. The density of each band was quantified using ImageJ version 1.8.0_112 (Windows version of NIH Image; downloaded from https://imagej.nih.gov/ij/download.html).

### Image acquirement and quantification analysis

Immunofluorescence was captured by a fluorescence microscope (BZ-X700). Quantification of immunofluorescence was performed using ImageJ version 1.8.0_112 using at least five lumbar spinal cord sections or five peripheral nerve sections for each animal in each group using the area fraction technique as previously described^47^.

### Electron microscopy

The animals were perfused intracardially with saline followed by cold 4% paraformaldehyde. The sciatic nerve was prefixed with a fixation buffer (2.5% glutaraldehyde, 0.1 M sucrose, 3 mM CaCl_2,_ and 0.1 M sodium cacodylate, pH 7.4) overnight at 4 °C. After being rinsed in PBS, the tissue was post-fixed with 1% osmium tetroxide for 2 h, dehydrated in ethanol and propylene oxide, and embedded in Epon resin (Epon 812 resin kit; TAAB Laboratories, Aldermaston, Berkshire, UK). Ultrathin Sects. (80 nm) were stained with uranyl acetate for 5 min and with lead acetate for 10 min and then examined with a transmission electron microscope (Tecnai 20; FEI Company Japan Ltd, Tokyo, Japan)^45^.

### Mononuclear cell isolation from the sciatic nerve and flow cytometric analysis

After the transcardial perfusion by ice-cold PBS, sciatic nerves were removed, minced with scissors, then suspended in RPMI media. Then, the minced pieces were further dissociated using a 1000-µl pipette. The cell suspension was passed into the FACS buffer through a 100-µm cell strainer. This step was repeated several times. The cell suspension was centrifuged at 800 × *g* at 4 °C for 5 min, the supernatant discarded, and the cell pellet resuspended in 1 ml FACS buffer^48^. For surface marker staining, cells were incubated with fluorochrome-conjugated antibodies against CD45, CD3, I-A/I-E, CD11b, and CD11c for 30 min at 4 °C and analyzed in a BD FACSVerse™ flow cytometer (Becton, Dickinson and Company, NJ). The percentage of CD45^+^ leukocytes, CD3^+^ T cells, I-A/I-E^+^ monocytes, CD11b^+^CD11c^−^ macrophages, and CD11b^+^CD11c^+^ phagocytes was measured.

### Cell culture

To analyze the phagocytic activity of monocytes in vitro, peripheral heparinized whole blood was isolated from mice. Then, 100 µl of whole blood was incubated with 10 µl of pHrodo Green *E. coli* BioParticles Conjugate (10 mg/ml; Thermo Fisher Scientific, MA) and 40 µl of Gibco RPMI 1640 medium (Thermo Fisher Scientific) at 37 °C and 5% CO_2_ for 30 min. After a washing step, the cells were analyzed by flow cytometry using a Sony SH-800 (Sony Corporation, Tokyo, Japan).

### Statistical analysis

Data are expressed as the mean ± standard error of the mean (SEM). Pairwise comparisons between two groups were performed using the unpaired *t *test and log-rank test. Comparisons between the three groups used in Fig. 2c were performed by two-way repeated-measures analysis of variance (ANOVA) followed by Bonferroni posttests. Multiple comparisons in Suppl. Figure 2 were performed by one-way factorial ANOVA. Survival time was compared using the Kaplan–Meier method and the log-rank test. A value of *p* < 0.05 was considered statistically significant. Quantitative analysis for FCM in Suppl. Figure 4b were performed by unpaired *t* test. All statistical analyses were conducted using JMP pro 12 software (SAS Institute, Cary, NC). Graphical images were built using PRISM 9 software (GraphPad Software, CA).

### Ethical approval

The handling and sacrifice of all animals were conducted according to the guidelines for the proper conduct of animal experiments published by the Science Council of Japan, as well as the ARRIVE (Animal Research: Reporting of In Vivo Experiments) guidelines for animal research. Ethical approval for the study was granted by the Animal Care and Use Committee of Kyushu University (#A30-051).

## Supplementary Information

Supplementary Information.
