# Supplementary material for: Clearance of peripheral nerve misfolded mutant protein by infiltrated macrophages correlates with motor neuron disease progression
Source: Sci Rep. 2021 Aug 12;11:16438. doi: 10.1038/s41598-021-96064-6 (PMC8360983; doi:10.1038/s41598-021-96064-6)
Supplement: Supplementary file 1 — Supplementary Information. [file 41598_2021_96064_MOESM1_ESM.pdf]

SUPPLEMENTARY TABLE 1. Antibodies used for immunohistochemistry.

**Primary antibody**	**Dilution**	**Source**
Rat monoclonal anti-ALS mutant SOD1	1:100	Merck, Darmstadt, Germany
Mouse monoclonal anti-arginase-1	1:200	Santa Cruz Biotechnology, Santa Cruz, CA, USA
Rabbit polyclonal anti-iNOS antibody	1:200	Bioss, Beijing, China,
Rat monoclonal anti- CCL2-antibody	1:100	Santa Cruz Biotechnology, Santa Cruz, CA, USA
Rabbit polyclonal anti-CD45 antibody	1:200	Abcam, Cambridge, UK
Rabbit polyclonal anti-Iba1 antibody	1:500	Wako, Osaka, Japan
Mouse monoclonal anti-phosphorylated neurofilament-H (SMI-31) antibody	1:100	Funakoshi, Tokyo, Japan
Mouse monoclonal anti-non phosphorylated neurofilament-H (SMI-32) antibody	1:100	Funakoshi, Tokyo, Japan
Mouse monoclonal anti-Schwann antibody	1:500	Cosmo Bio Company, Tokyo, Japan
Rabbit mouse monoclonal anti-neuronal nuclei (NeuN) antibody	1:100	Merck, Darmstadt, Germany

SUPPLEMENTARY TABLE 2. Antibodies used for flow cytometry.

**Primary antibody**	**Dilution**	**Source**
PE-conjugated Mouse monoclonal anti-I-A/I-E antibody	1:30	BioLegend, San Diego, CA, USA
APC-conjugated Mouse monoclonal anti-CD11c antibody	1:30	eBioscience, San Diego, CA, USA
PerCP/Cyanine5.5 Mouse monoclonal anti-CD3 antibody	1:30	BioLegend, San Diego, CA, USA
PE/Cy7-conjugated Mouse monoclonal anti-CD11b antibody	1:30	BioLegend, San Diego, CA, USA
FITC-conjugated Rat monoclonal anti-CD45 antibody	1:30	Invitrogen, Carlsbad, CA, USA

SUPPLEMENTARY FIGURE 1

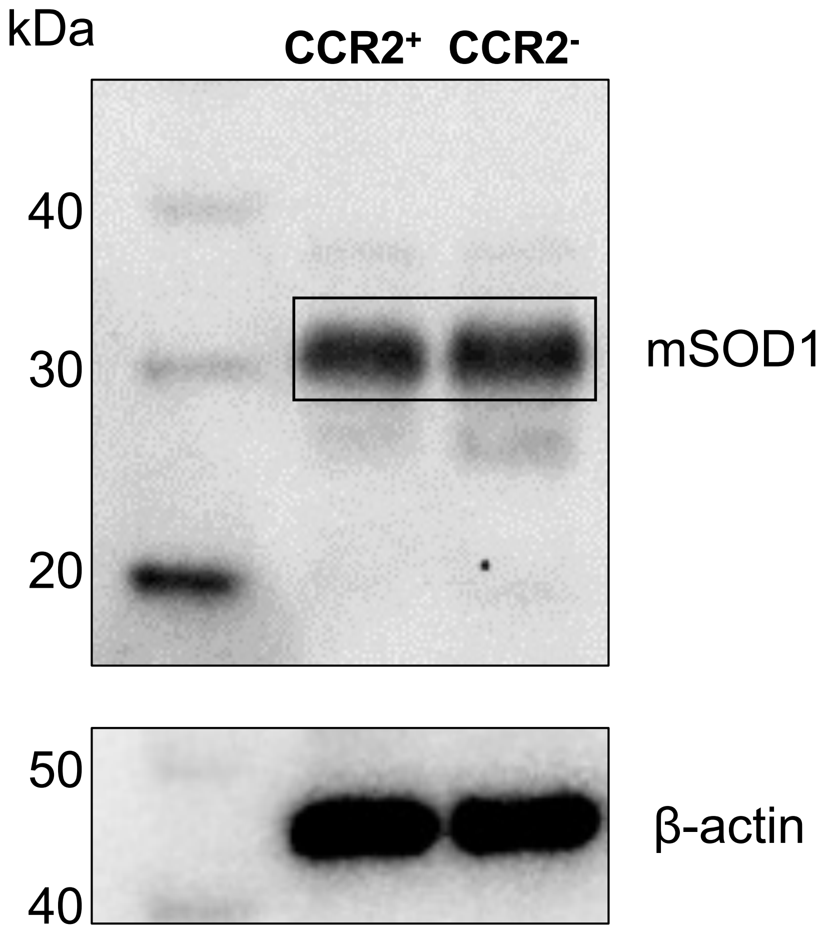

SUPPLEMENTARY FIGURE 2

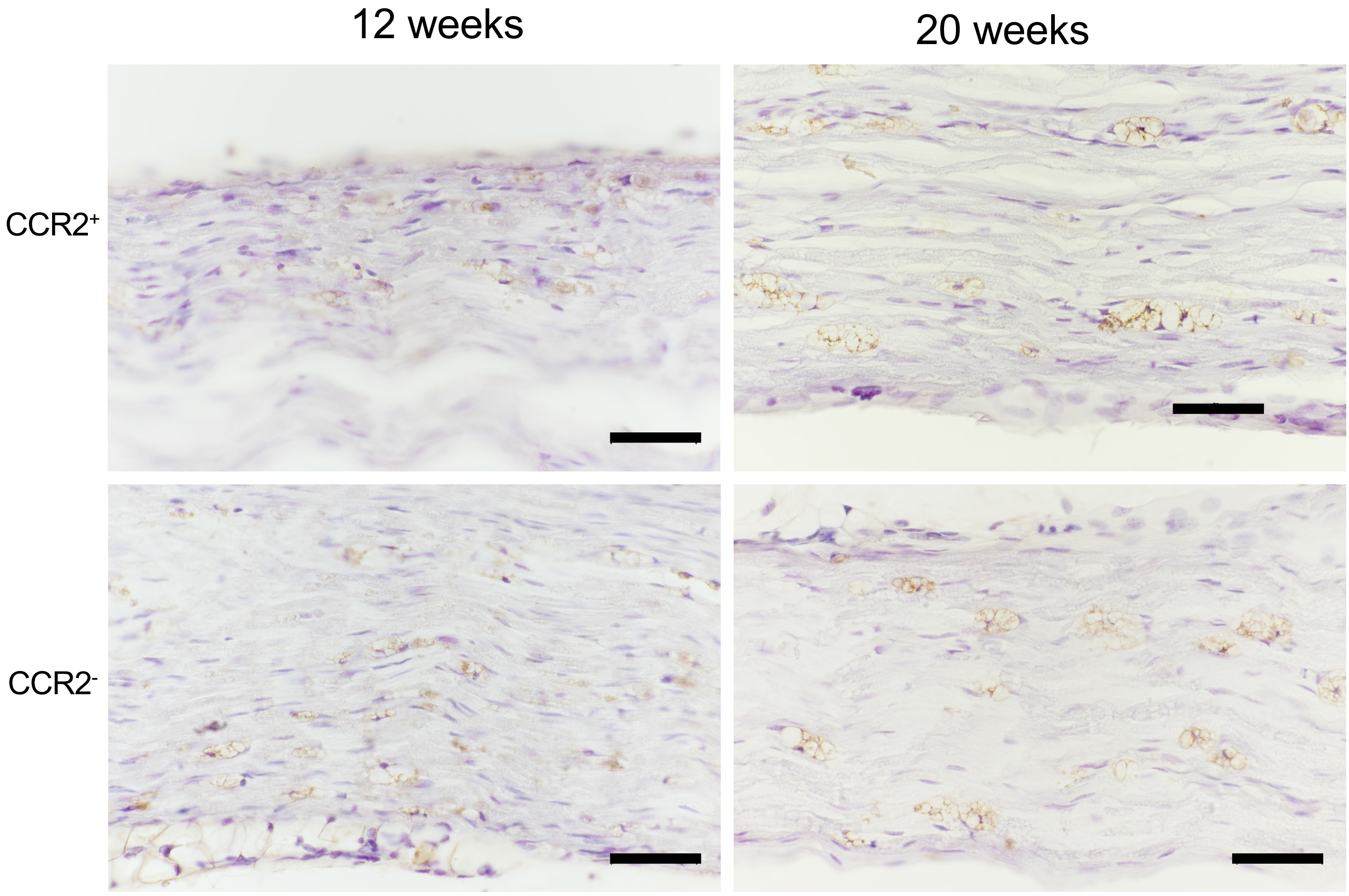

SUPPLEMENTARY FIGURE 3

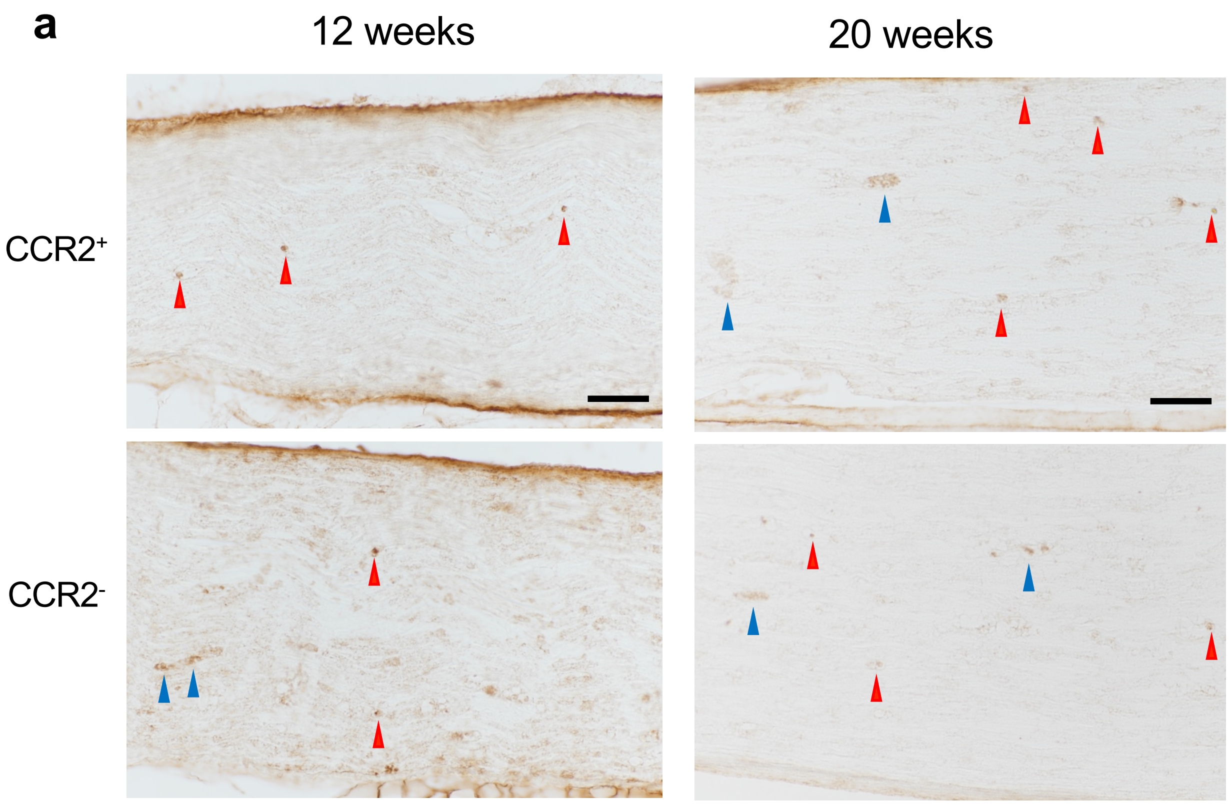

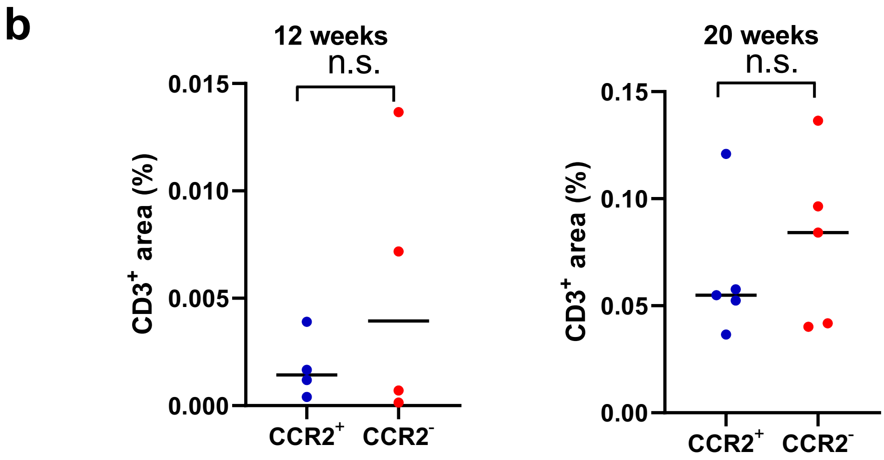

SUPPLEMENTARY FIGURE 4

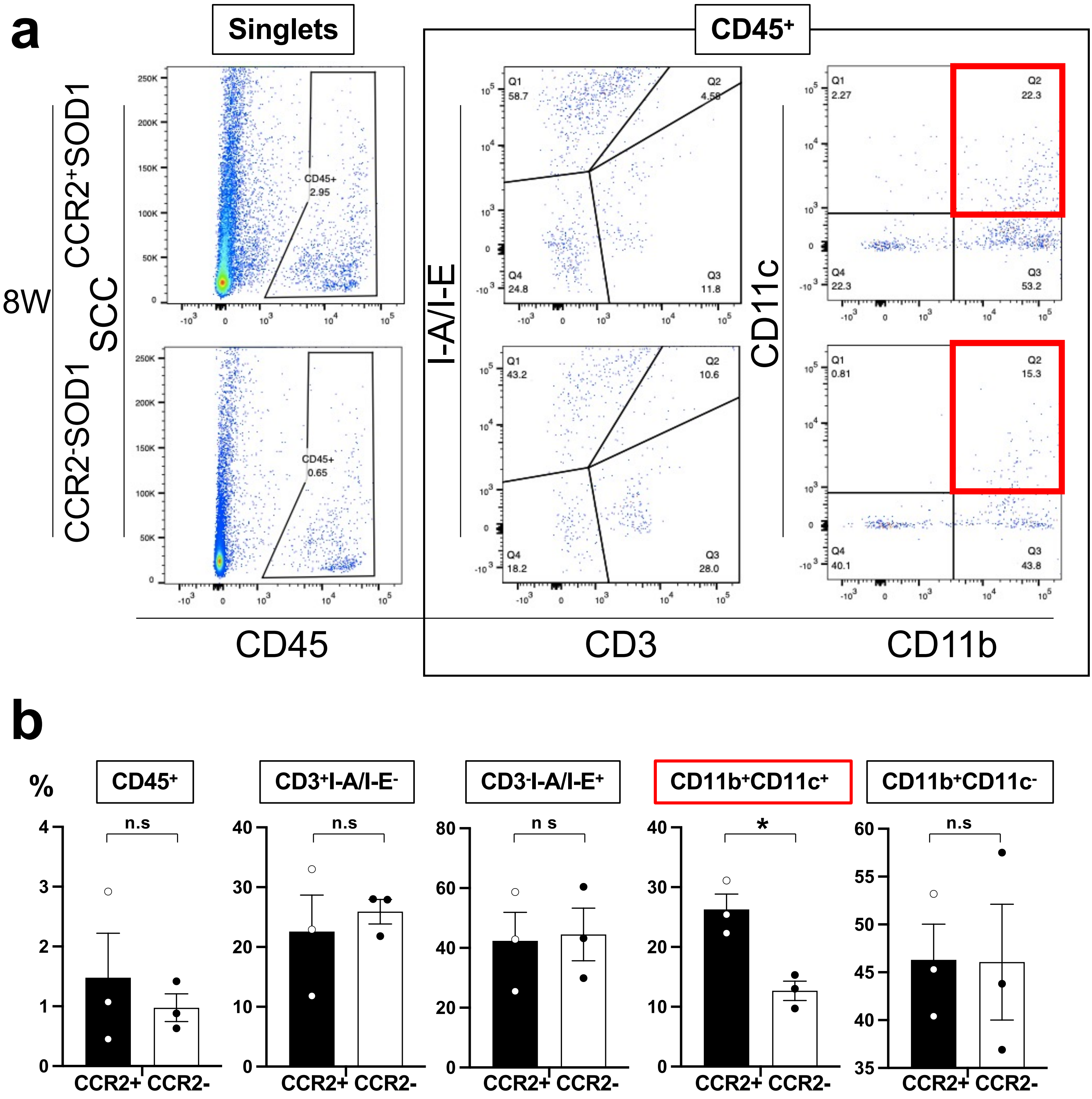

SUPPLEMENTARY FIGURE 5

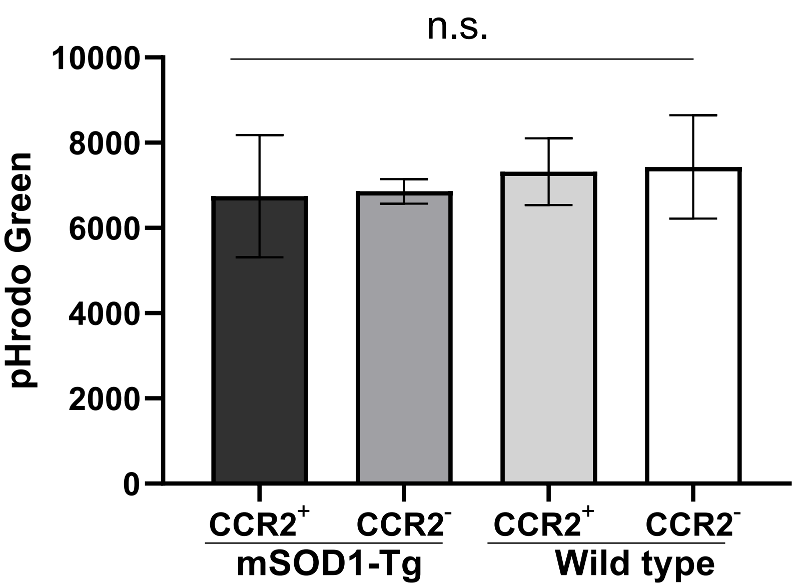

SUPPLEMENTARY FIGURE 6

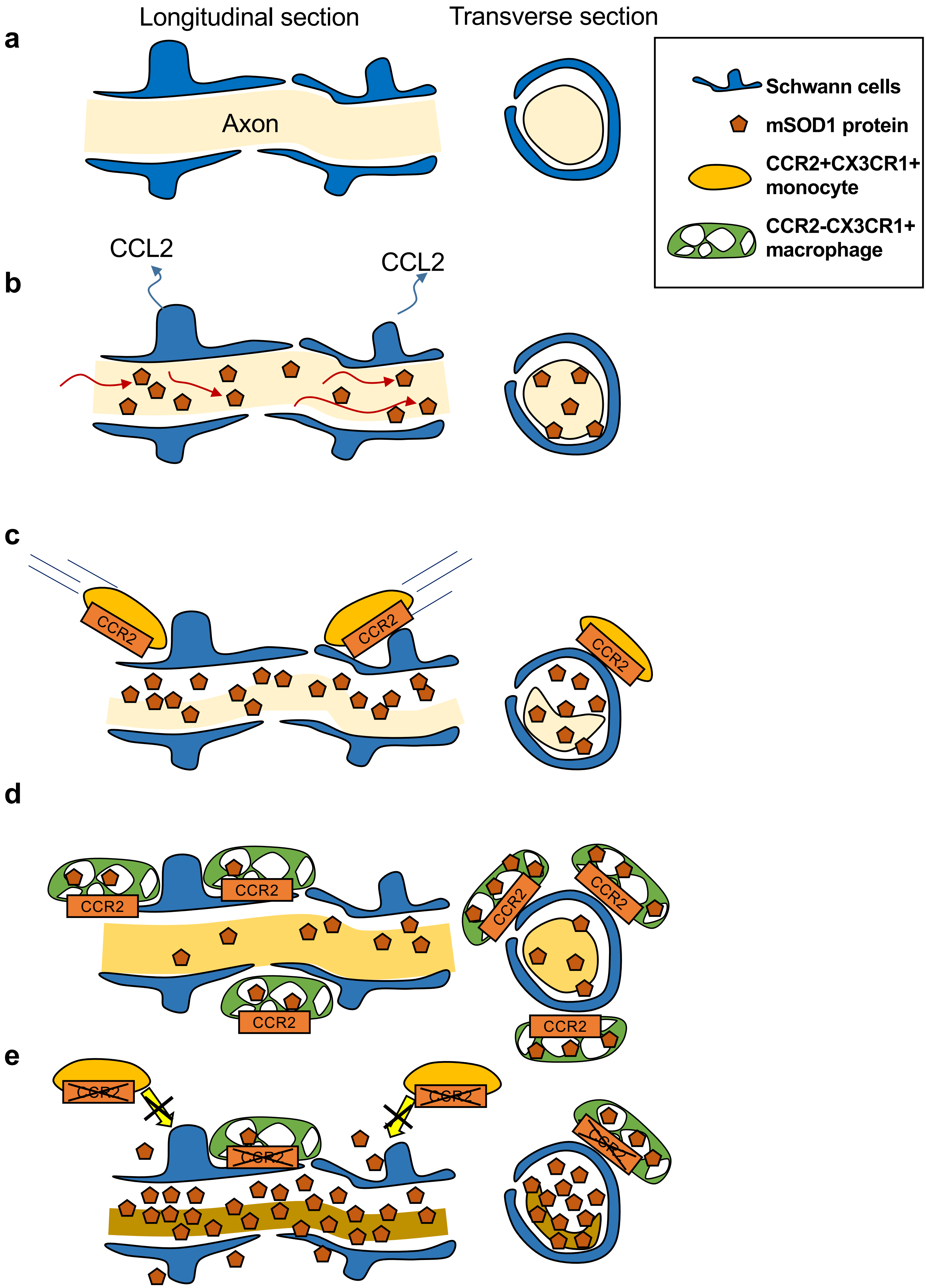

SUPPLEMENTARY FIGURE 7

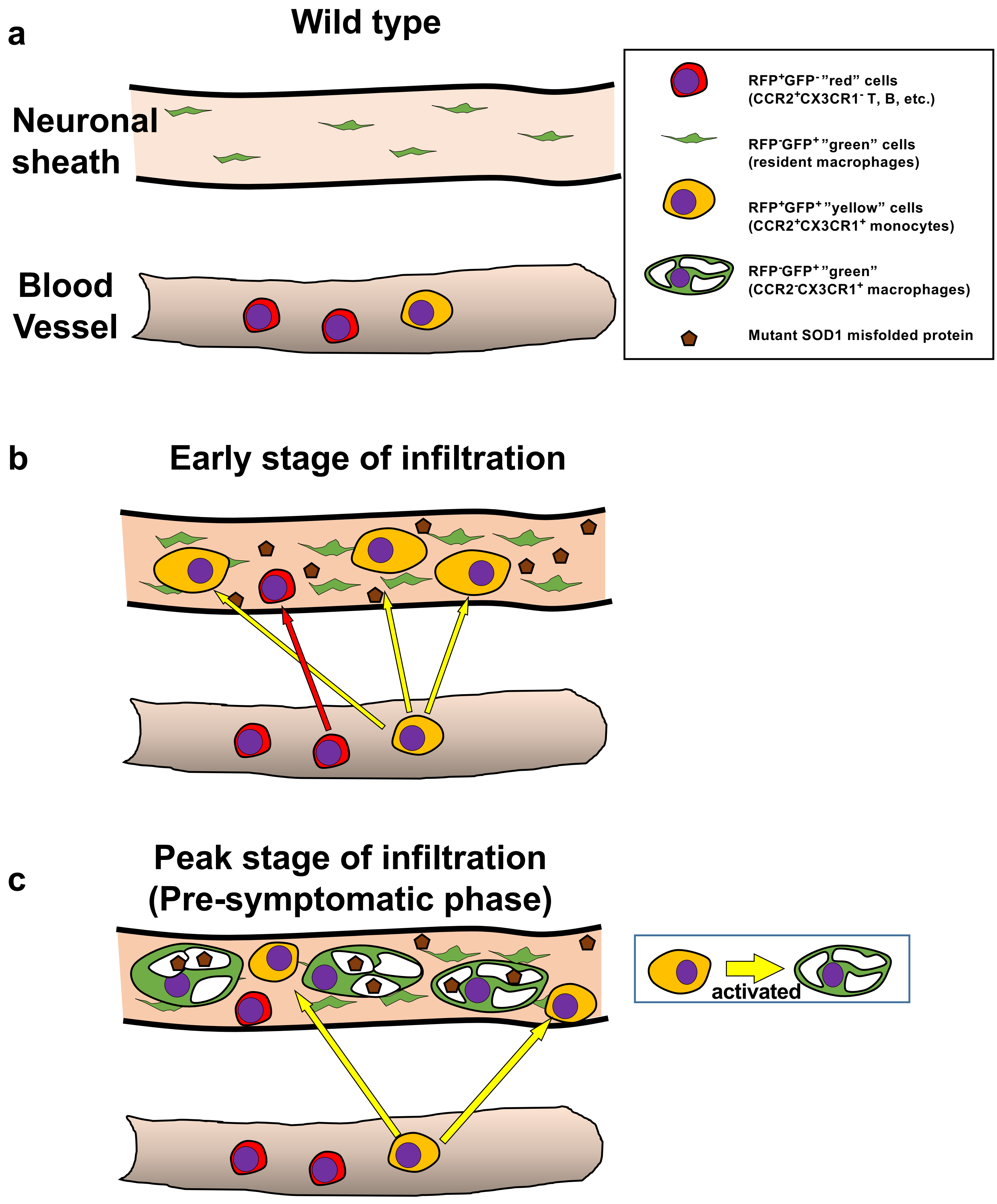

SUPPLEMENTARY FIGURE 8

a

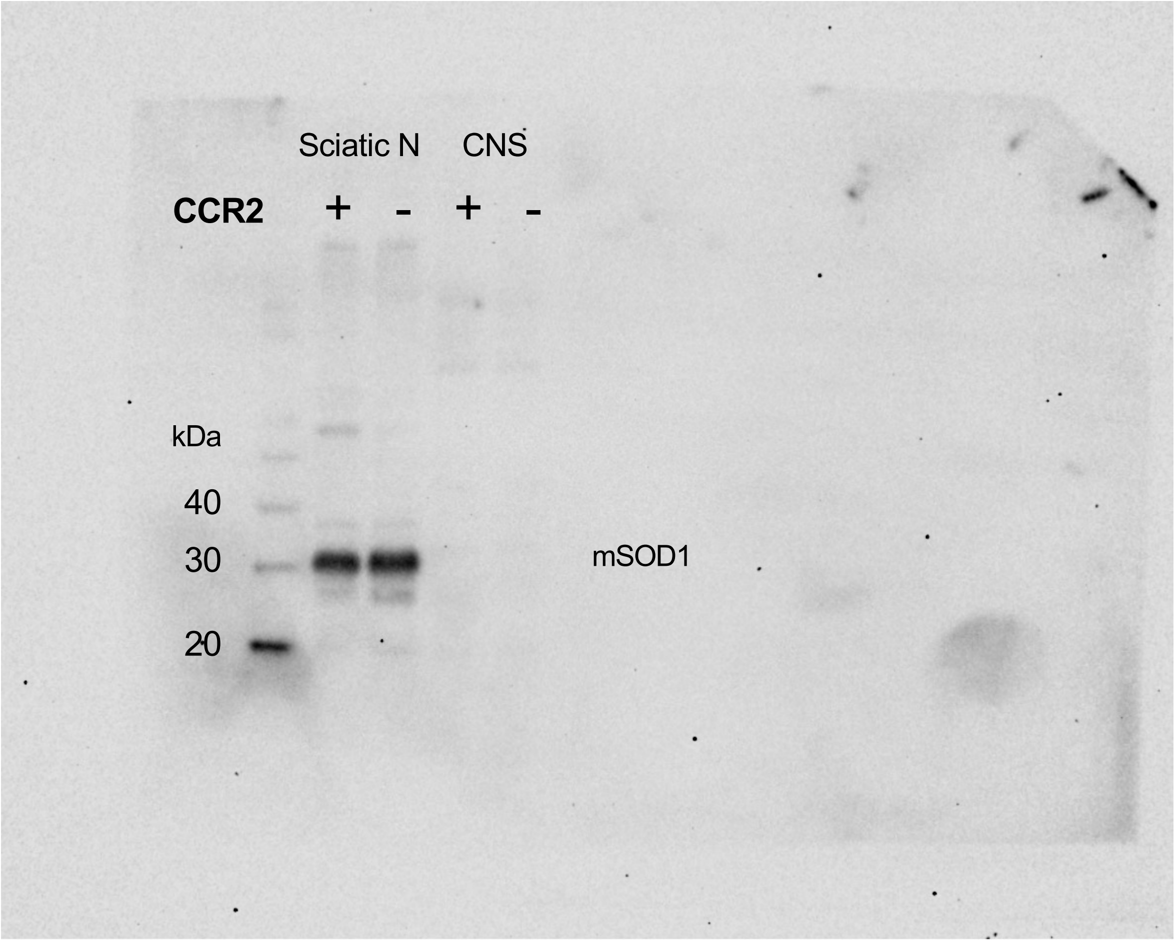

b

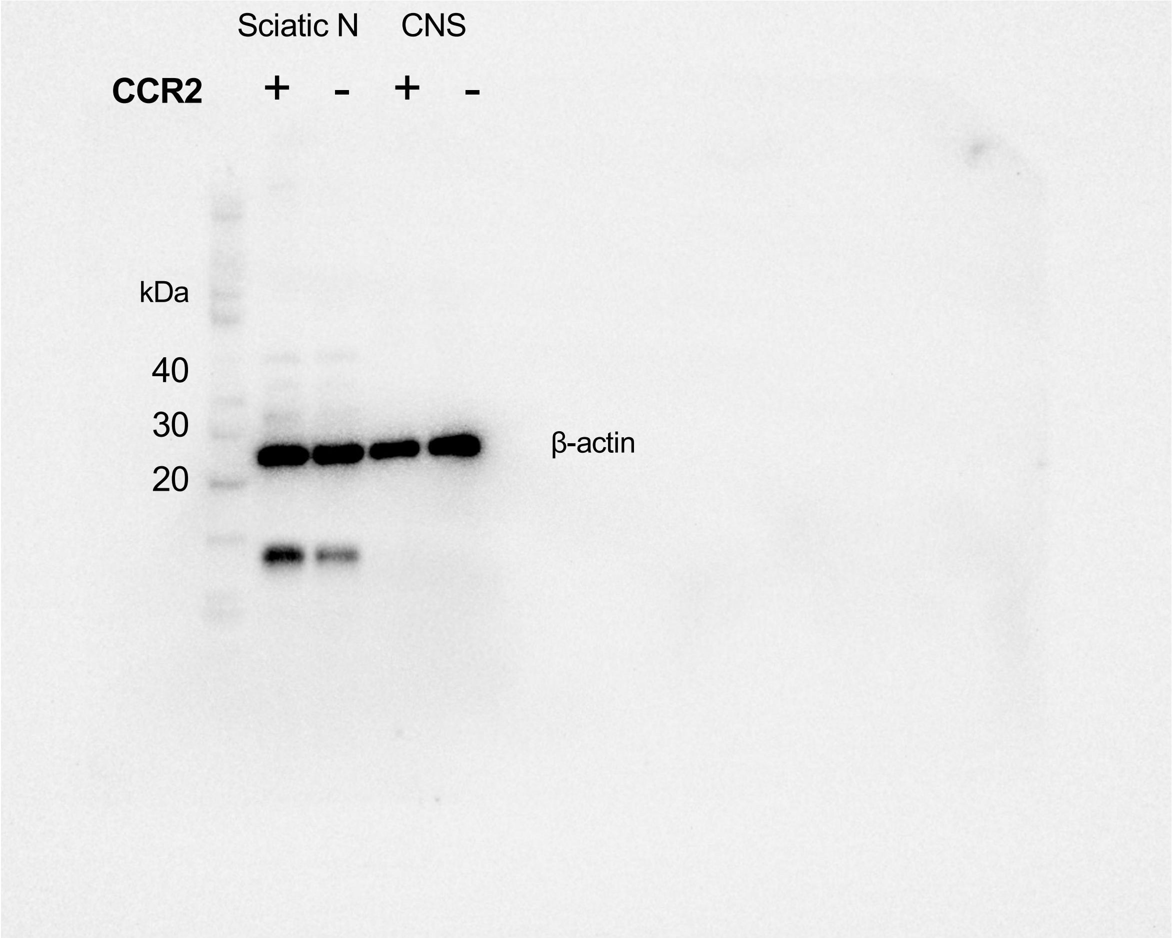

SUPPLEMENTARY FIGURE LEGENDS

SUPPLEMENTARY FIGURE 1: Western blotting confirms the aggravation of mSOD1 accumulation in the sciatic nerve by CCR2 ablation.

Western blotting of mSOD1 in the sciatic nerves of CCR2-deficient *SOD1*^{G93A} mice and CCR2-positive littermates. Sciatic nerve samples were pooled from three mice per group. Accumulation of mSOD1 protein was increased in CCR2-deficient *SOD1*^{G93A} mice compared with CCR2-positive *SOD1*^{G93A} mice at 12 weeks of age. Quantification of western blot analysis of mSOD1 protein levels normalized to β-actin levels were 1.06 for CCR2-deficient *SOD1*^{G93A} mice and 0.87 for CCR2-positive littermates.

SUPPLEMENTARY FIGURE 2: Immunostaining for CD68-positive cells in the sciatic nerve. CCR2-positive and CCR2-deficient *SOD1*^{G93A} mice showed CD68-positive foamy macrophages in the sciatic nerve. Scale bar: 50 µm.

SUPPLEMENTARY FIGURE 3: Immunostaining for CD3^{+} T cells in the sciatic nerve. (a) CCR2-positive and CCR2-deficient *SOD1*^{G93A} mice showed rare CD3^{+} T cell infiltration into the peripheral nerve. Red arrowheads indicate infiltrated T cells. Blue arrowheads indicate non-specific staining in foamy macrophages. (b) There was no

difference in $CD3^+$ area (%) between the two mouse genotypes at 12 and 20 weeks of age ($p = 0.308$, n = 4 and $p = 0.521$, n = 5). The horizontal line represents the mean value. Scale bar: 50 µm. n.s. = not significant.

SUPPLEMENTARY FIGURE 4: Representative flow cytometry plots and quantitative analysis.

(a) Representative plots showing the gating strategy for isolated immune cells from the sciatic nerve tissues of 8 w mSOD1-Tg mice with each experimental group. After the gating for $CD45^+$ cells in the singlet population, $CD45^+$ cells were further gated by CD3 and I-A/I-E, and by CD11b and CD11c. Red rectangles in the right column indicate gates for $CD11b^+/CD11c^+$.

(b) Percentages of designated cell populations in each experimental group ($CD45^+$ leukocytes in singlet gate, $CD3^+$I-A/I-E^- T cells in $CD45^+$ gate, $CD3^-$I-A/I-E^+ monocytes lineage cells in $CD45^+$ gate, $CD11b^+CD11c^-$ macrophages in $CD45^+$ gate, and $CD11b^+/CD11c^+$ differentiated macrophages in $CD45^+$ gate). Bar graphs indicate the mean ± SEM. n = 3 for each group. *: $p < 0.05$, n.s: not significant.

SUPPLEMENTARY FIGURE 5: Phagocytic activity of peripheral blood

macrophages. The graph shows the phagocytosis of *Escherichia coli* by macrophages. No significant difference in phagocytic activity was observed among *CCR2*$^{RFP/RFP}$ *SOD1*^{G93A} mice, *CCR2*$^{RFP/WT}$ *SOD1*^{G93A} mice, *CCR2*$^{RFP/RFP}$ mice, and *CCR2*$^{RFP/WT}$ mice. A one-way analysis of variance analysis was used for the statistical comparison. Data are represented as the mean ± SEM. n.s. = not significant.

SUPPLEMENTARY FIGURE 6: Schema showing mSOD1 protein clearance from the peripheral nerves by macrophages infiltrated from the peripheral blood.

(a) Normal peripheral nerve. (b) Accumulation of mSOD1 protein produced in the anterior horn cell and carried by axonal transport in the peripheral nerve axons of *SOD1*^{G93A} mice promotes CCL2 secretion by Schwann cells. (c) CCL2 attracts CCR2^{+} peripheral blood monocytes to the peripheral nerve. (d) In CCR2-positive *SOD1*^{G93A} mice, peripheral blood-borne CCR2^{+} monocytes phagocytose mSOD1 protein with a foamy appearance and contribute to the clearance of mSOD1 protein from the peripheral nerve. (e) Conversely, in CCR2-deficient *SOD1*^{G93A} mice, insufficient infiltration of peripheral blood monocytes lacking CCR2 into the peripheral nerve results in impaired mSOD1 protein clearance, facilitating mSOD1 accumulation in the peripheral nerve, which accelerates axonal deformation and lower motoneuron death.

CCR2 is also expressed on protective T cells and other cells, which indicates that these protective cells may also be involved.

SUPPLEMENTARY FIGURE 7: Schema showing the transformation of CCR2 and CX3CR1 expressions on $CCR2^{+}$ monocytes from the peripheral blood.

(a) In the wild-type mouse peripheral nerve, $CCR2^{+}CX3CR1^{-}$ (red) cells (T and B cells, basophils) and $CCR2^{+}CX3CR1^{+}$ (yellow) cells (monocytes) are in the idle status in the blood or spleen. $CCR2^{-}CX3CR1^{+}$ (green) resident macrophages are in the nerve sheath.

(b) At the early stage of infiltration, the numbers of $CCR2^{+}CX3CR1^{+}$ (yellow) cells (monocytes) and $CCR2^{+}CX3CR1^{-}$ (red) cells increases in the nerve sheath.

(c) After monocytes infiltrate the peripheral nerves, CCR2 expression is reduced as well as their activation and differentiation into foamy macrophages, which results in an increase in green cell numbers.

SUPPLEMENTARY FIGURE 8: Western blotting confirms the accelerated accumulation of mSOD1 in the sciatic nerve than in the CNS.

(a) Full-size images of western blotting of mSOD1 in the sciatic nerve and CNS of CCR2-deficient *SOD1*^{G93A} mice and CCR2-positive littermates (12 weeks of age). Note

that mSOD1-positive bands are only visible in the sciatic nerve samples but not in the CNS samples, which indicates proceedings of misfolded protein accumulation in the sciatic nerve than in the CNS.

(b) Control experiments for (a), using anti-β-actin antibody.

Sciatic nerve and CNS samples were pooled from three mice per group.

Cropped images are presented as Suppl Fig. 1.
